# The GFPT2-O-GlcNAcylation-YBX1 axis promotes IL-18 secretion to regulate the tumor immune microenvironment in pancreatic cancer

**DOI:** 10.1038/s41419-024-06589-7

**Published:** 2024-04-04

**Authors:** Hui-Ru Zhang, Tian-Jiao Li, Xian-Jun Yu, Chen Liu, Wei-Ding Wu, Long-Yun Ye, Kai-Zhou Jin

**Affiliations:** 1 Department of Pancreatic Surgery, Fudan University Shanghai Cancer Centre, Shanghai, China; 2 Department of Oncology, Shanghai Medical College, Fudan University, Shanghai, China; 3 Shanghai Pancreatic Cancer Institute, Shanghai, China; 4 Pancreatic Cancer Institute, Fudan University, Shanghai, China

**Keywords:** Cancer, Cancer microenvironment

## Abstract

The immunosuppressive microenvironment caused by several intrinsic and extrinsic mechanism has brought great challenges to the immunotherapy of pancreatic cancer. We identified GFPT2, the key enzyme in hexosamine biosynthesis pathway (HBP), as an immune-related prognostic gene in pancreatic cancer using transcriptome sequencing and further confirmed that GFPT2 promoted macrophage M2 polarization and malignant phenotype of pancreatic cancer. HBP is a glucose metabolism pathway leading to the generation of uridine diphosphate N-acetylglucosamine (UDP-GlcNAc), which is further utilized for protein O-GlcNAcylation. We confirmed GFPT2-mediated O-GlcNAcylation played an important role in regulating immune microenvironment. Through cellular proteomics, we identified IL-18 as a key downstream of GFPT2 in regulating the immune microenvironment. Through CO-IP and protein mass spectrum, we confirmed that YBX1 was O-GlcNAcylated and nuclear translocated by GFPT2-mediated O-GlcNAcylation. Then, YBX1 functioned as a transcription factor to promote IL-18 transcription. Our study elucidated the relationship between the metabolic pathway of HBP in cancer cells and the immune microenvironment, which might provide some insights into the combination therapy of HBP vulnerability and immunotherapy in pancreatic cancer.

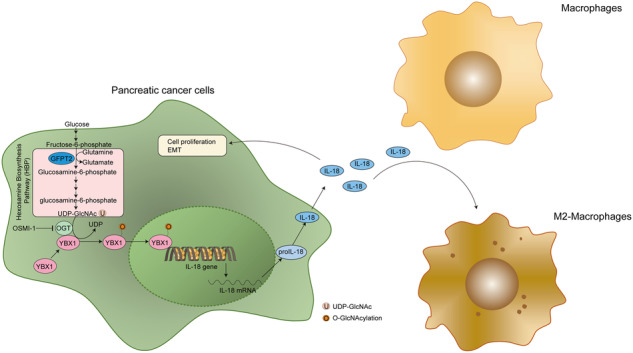

## Introduction

Pancreatic cancer ranks as the third leading cause of cancer-related death in the United States [1]. Pancreatic ductal adenocarcinoma (PDAC) accounts for 90% of pancreatic cancer cases [2]. For all stages combined, the 5-year relative survival rate for pancreatic cancer is only 11% [3]. Therefore, decoding the causes of malignant progression of PDAC is very important for improving the prognosis of patients.

Immunotherapy has shown significant benefits in solid organ tumors and immune checkpoint blockade has improved treatment options for various cancers, such as metastatic melanoma, renal cell carcinoma, colorectal cancer with microsatellite instability, non-small-cell lung cancer and Hodgkin’s lymphoma [4–7]. However, the efficacy of immunotherapy in treating PDAC has been disappointing. Fortunately, combination immunotherapy strategies targeting the immunosuppressive tumor microenvironment, which is formed by several intrinsic and extrinsic mechanisms in PDAC, have become a valuable treatment option [8].

In this study, we aimed to identify immune-related prognostic genes and further explored the communication mechanism between tumor cells and the immune microenvironment. We performed transcriptome sequencing on clinical specimens. We identified glutamine-fructose-6-phosphate transaminase 2 (GFPT2) as an immune-related prognostic gene in PDAC. In live tumor cells, GFPT2 overexpression promoted the excessive use of glutamine by tumor cells and competitively impaired the uptake of glutamine by macrophages. Eventually, mitochondrial fission and phagocytosis were impaired in macrophages [9]. In non-small cell lung cancer, KRAS/LKB1 co-mutant cancer cells showed high levels of hexosamine biosynthesis pathway (HBP) metabolites, higher flux through the HBP and elevated dependence on the HBP enzyme GFPT2. More importantly, targeting GFPT2 reduced KRAS/LKB1 co-mutant tumor cell growth in in vitro and in vivo models [10]. In PDAC, GFPT2 was reported to promote cell motility and glucose uptake via the HBP [11]. However, the effects of GFPT2 on the immune microenvironment in PDAC is unknown.

Here, we demonstrated that GFPT2 regulated O-GlcNAcylation modifications in tumor cells and then promoted macrophage M2 polarization and the malignant phenotype of PDAC. We identified IL-18 as a key downstream target of GFPT2 in regulating the immune microenvironment via proteomics. We confirmed that YBX1 was O-GlcNAcylated and translocated to the nucleus by GFPT2-mediated O-GlcNAcylation. Our study is the first to report the role of GFPT2 in the regulation of the immune microenvironment in PDAC, which might provide some insights into the combination therapy of HBP vulnerability and immunotherapy in PDAC.

## Methods

### Patient population

We enrolled 22 patients who underwent surgical treatment at the Fudan University Shanghai Cancer Center (FUSCC) between April 2013 and May 2020 and who were pathologically confirmed to have PDAC. According to the tumor-node-metastasis staging system of the 8th edition of the American Joint Committee on Cancer, the clinical stages of these patients ranged from IB to IIB [12]. All patient information, clinicopathological characteristics and overall survival time were acquired from medical records and telephone interviews. Overall survival time was determined from the date of surgery to the date of death or the end time of follow-up.

### Cell culture and reagents

The human PDAC cell lines PANC-1, CFPAC-1 and SW1990 were purchased from American Type Culture Collection (ATCC) and were further authenticated by short tandem repeat (STR) analysis. PANC-1 and SW1990 cells were cultured in Dulbecco’s modified Eagle’s medium (DMEM) and CFPAC-1 cells were cultured in Iscove’s modified Dulbecco’s medium (IMDM). All media were supplemented with 10% fetal bovine serum (FBS) (ExCell Bio, cat. FSP500, China) and 1% penicillin-streptomycin solution in a humidified atmosphere of 5% CO2 and 95% air at 37 °C. Actinomycin D (cat. SBR00013) was purchased from Sigma. OSMI-1 (cat. S983501) was purchased from selleck.cn.

### Extraction of CD14+ monocytes

Extraction of CD14+ monocytes from fresh human peripheral blood mononuclear cells (PBMCs) was performed using the EasySep™ Human CD14 Positive Selection Kit II (Catalog #17858) and strictly followed the instructions. After isolation, the CD14+ monocytes were stimulated with 25 ng/ mL recombinant human M-CSF (PeproTech, USA, 300-25) for 7 days to get macrophages.

### Western blotting

Western blotting was performed using antibodies against GFPT2 (1:1000, ab190966, Abcam), YBX1 (1:1000, A3534, ABclonal), IL-18 (1:2000, 10663-1-AP, Proteintech), O-GlcNAcylation (1:1000, ab2739, Abcam), OGT (1:1000, #24083, Cell Signaling Technology), E-cadherin (1:1000, ab40772, Abcam), vimentin (1:1000, ab92547, Abcam) and beta actin (1:1000, ab8226, Abcam).

### Immunohistochemistry (IHC) and multiplex immunohistochemistry (mIHC)

Briefly, paraffin-embedded tissue slides underwent deparaffinization, rehydration, antigen retrieval, removal of endogenous peroxidase, blocking with 5% BSA and incubation with primary antibodies. Antibodies against IL-18, GFPT2, YBX1, E-cadherin, and N-cadherin were used at a concentration of 1:200. Then the slides were incubated with tyramide-conjugated fluorophore and the nuclei were stained with DAPI for mIHC. For IHC, the procedure was similar to that for mIHC. Secondary antibodies were conjugated with enzymes and diaminobenzidine as the chromogen and Mayer’s hematoxylin as the counterstain.

### 5-Ethynyl -2′- deoxy uridine (EdU) cell proliferation assay

Cell proliferation capacity was assessed using the EdU cell proliferation kit (C0078S, Beyotime). The procedure was carried out according to the instructions of the kit. The working concentration of EdU was 10 μM.

### Transwell cell migration assay

Pancreatic cells suspended in serum-free medium were added to the upper chamber (8 μm pore size; Millipore) and medium containing 20% fetal bovine serum was added to the lower chamber for the coculture system. The incubation times for PANC-1, SW1990 and CFPAC-1 cells were 10 h for 100,000 cells, 24 h for 200,000 cells and 24 h for 200,000 cells, respectively. Then, the upper chamber was fixed with 4% paraformaldehyde and stained with 0.1% crystal violet. Cancer cells that passed through the polycarbonate membrane were photographed using a Leica microscope. The assessment of the relative migration capacity of cancer cells was performed by averaging cell counts from five different fields of view.

### Colony formation assay

Pancreatic cancer cells were seeded into 6-well plates (CFPAC-1 was 2000 cells per well, SW1990 and PANC-1 was 1000 cells per well) in triplicate and cultured for 14 days. The culture was refreshed every three days. Finally, colonies were fixed with 4% paraformaldehyde solution and stained with 0.1% crystal violet solution. Then, the number of colonies counted under a microscope. Representative results of three independent experiments with consistent trends were presented.

### Mass spectrometry (MS) quantification and co-immunoprecipitation (Co-IP) LC-MS/MS analysis

Label-free quantitative mass spectrometry was used to detect GFPT2-regulated proteins in this study. Briefly, cells were first subjected to cleavage to measure the concentration and then underwent precipitation and enzymatic hydrolysis. Mass spectrometry analysis was performed using Orbitrap Fusion Lumos (Thermo Scientific, USA) after desalting. The data were analyzed with Proteome Discover 2.4 (Sequent HT) (Thermo Scientific, USA). For Co-IP/MS, the Co-IP assay was performed using an antibody against O-GlcNAcylation in SW1990 cells. Immunocomplexes were subjected to SDS-PAGE separation. Then, the gels were stained with a silver staining kit (Beyotime, China) and cut off for LC-MS/MS analysis using Q Exactive Plus (Thermo Scientific, USA). MaxQuant 2.0.1.0 was used to search the mass spectrometry database. The following protein data were used: UniProt-Reference proteome - *Homo sapiens* (Human) [9606]-81791-20230317. Fasta, which comes from https://www.uniprot.org/proteomes/UP000009606 protein database. After mass spectrum data retrieval, PSM FDR ≤ 0.01 and protein FDR ≤ 0.01 were used as the standards for peptide, site and protein identification.

### Primers and quantitative real-time PCR (qRT‒PCR)

Primer for GFPT2: 5′- AGGATCCTTGCTTCGCCAAA -3′ (forward), 5′- GTATAATGGGG CGACCCTGG -3′ (reverse). Primer for IL-18: 5′- GCAGTCTACACAGCTTCGGG -3′ (forward), 5′- GCAAAGAGCCATCTGCGACA -3′ (reverse). Total RNA of cells was extracted using TRIzol reagent (Invitrogen, USA). Then, cDNA was obtained by reverse transcription using a Vazyme HiScript III RT SuperMix for qPCR (+gDNA wiper) reagent kit. Finally, qRT-PCR was conducted by an ABI 7900HT Real-Time PCR system (Applied Biosystems, USA).

### Chromatin Immunoprecipitation (ChIP)

ChIP assays were performed using an antibody against YBX1 according to the instructions of a ChIP kit (#9005, Cell Signaling Technology). Primers from 1-6 were as follows: (1) 5′- GCTCAGCAAGCTGGGGAGAG -3′ (forward), 5′- AGAAGTACTCGTATCCTCTG -3′ (reverse); (2) 5′- CCGCCTTCAAACAGGCTCT -3′ (forward), 5′- GAGCCCCAACTTTTACGGAAG -3′ (reverse); (3) 5′- GCTCTGTGCCTTCCAAAACC -3′ (forward), 5’-TGCTGAAGTGTGACCAGG AAG-3′ (reverse); (4) 5′- GTATCAGATGCAAGCCACACG -3′ (forward), 5′- TTTGGTATCC CTCTCCCCAAG -3′ (reverse); (5) 5′- AAGGGAATTAGCAAGGTGGCA -3′ (forward), 5′- TCCCTGACTCTAGGAACCCC-3′ (reverse); (6) 5′- TCAGATGGACAACCACCTGTT -3′ (forward), 5′- GGGGCACAGGTGAGATAAGTT -3′ (reverse).

### Plasmids and RNA Interference

The coding sequences of human GFPT2 or YBX1 were cloned and inserted into the lentiviral vector pCDH-CMV-MCS-EF1-puro (SBI, USA) to generate GFPT2 or YBX1 expression plasmids and then lentivirus-infected pancreatic cancer cells. For shRNA, pLKO.1 puro was used to generate GFPT2 knockdown pancreatic cancer cells. The sequences of shGFPT2 were as follows: shGFPT2#1: 5′- GGTTGAACTTGCTAGTGAT -3′; shGFPT2#2: 5′- AGGTAACTTCAGTGCGTTTAT -3′. For IL-18 and YBX1 interference, the target sequences of the siRNAs were as follows: siIL-18#1: 5′- GAAUCUAAAUUAUCAGUCA -3′; siYBX1#1: 5′- GGAGGCAGCAAATGTTACA -3′. Pancreatic cancer cells were transfected with siRNAs using Lipofectamine 3000 reagent (Life Technologies, Invitrogen) according to the manufacturer’s instructions.

### Flow cytometry analysis of M2 macrophages

Immunocytes were first incubated with anti-CD16/32 antibody for 10 min at room temperature to block Fc receptors and subsequently stained with an antibody cocktail of surface markers for 30 min at 4 °C. Then, Fixable Viability Dye 780 (Thermo Fisher, 65-0865-14) was applied to the staining buffer for 10 min at 4 °C. The following antibodies were used for the human specimens: anti-CD45-APC, anti-CD11b-FITC, anti-CD206-PC5.5 and anti-CD163-PE. All antibodies were purchased from BioLegend. Finally, prepared samples were analyzed by flow cytometry (BD Fortessa). For human tumor specimens and mouse tumor specimens, the above flow analysis was performed after the specimen undergoing digestion, removal of red blood cells, and other steps.

### Detection of IL-18

Detection of IL-18 secreted by tumor cells was performed by an ELISA kit (ab215539, Abcam, UK). Cells were collected and lysed to measure the protein concentration and cell culture media were collected and centrifuged at 2000 × g for 10 min to remove debris. IL-18 assays were performed according to the manufacturer’s instructions. Unit protein concentration was used to correct for IL-18 secretion.

### Animal studies

BALB/c-nu mice were used in the tumorigenicity assay of GFPT2-altered pancreatic cancer cells. Briefly, 1 × 10^7 cells were subcutaneously grown in mice. Each group was repeated five times. After tumor formation, the tumor size was observed and recorded every 2 or 3 days. The experiment was terminated before the tumor volume of the mice reached 1500 mm^3^. Then, tumors were removed, photographed for documentation, and embedded in paraffin for IHC and IF assays. Similarly, C57BL/6 mice were used to detect the effects of GFPT2 on macrophage polarization and assess the effects of OSMI-1 on this axis. 2 × 10^6 cells were subcutaneously grown in C57BL/6 mice. The experiment procedure is the same as above. After the tumors were removed, a portion of the tumor was digested and followed by flow cytometric analysis of M2 macrophages.

### Statistical analysis

Experiments were repeated at least three times. All data in this study are presented as the mean ± SD. Two-tailed Student′s t-test was used to compare parameters between two groups, and the chi-square test was used to assess the correlation between two categorical variables. The Kaplan-Meier survival function and log-rank test were used to plot the survival curve and evaluate the overall survival of patients. The statistical significance of differences between groups was analyzed by GraphPad Prism 8 software. Differences were considered significant at *, *P* < 0.05; **, *P* < 0.01; ***, *P* < 0.001.

## Results

### GFPT2 was an immune-related prognostic gene in PDAC

**Fig. 1:**
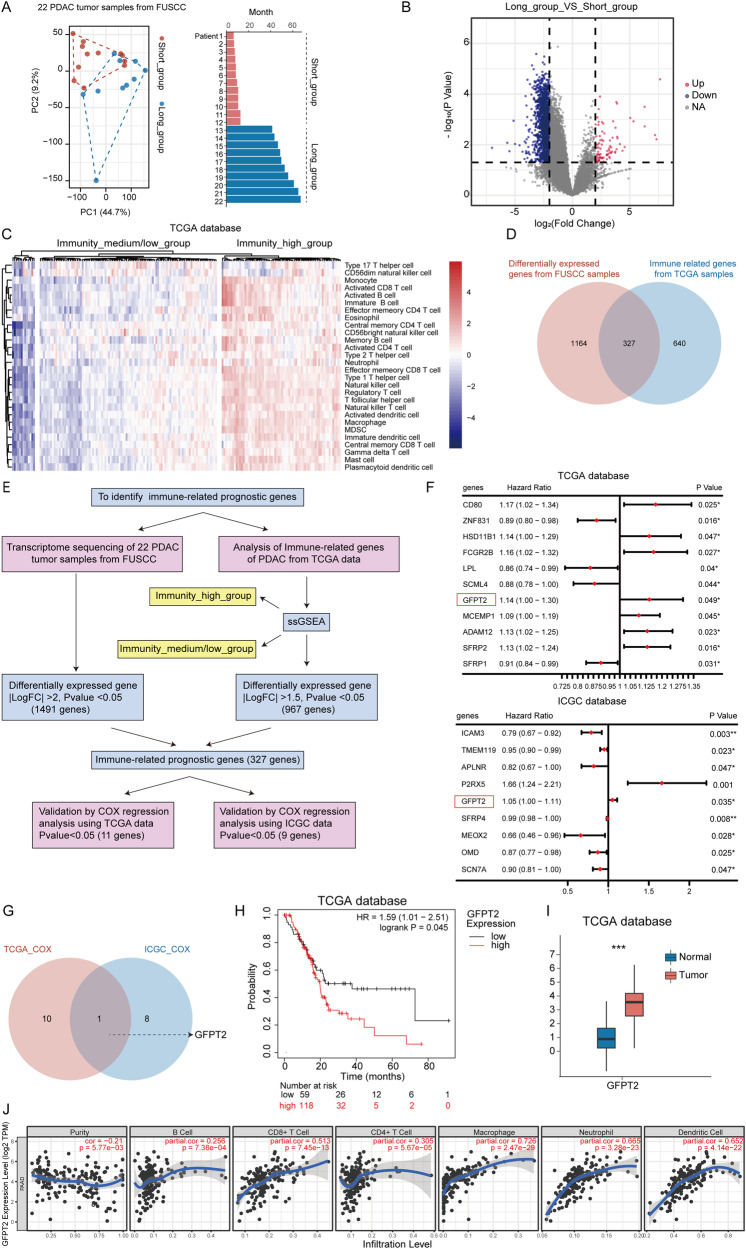
GFPT2 was an immune-related prognostic gene in pancreatic cancer. **A** Principal component analysis showed transcriptome sequencing sample features of 22 pancreatic ductal adenocarcinoma (PDAC) patients from FUSCC, and the bar chart showed the survival time of the patients. **B** Volcano map showing differences in gene expression between groups with an overall survival of less than 1 year and those with an overall survival of more than 3 years (fold change > 4 or < -4). **C** Heatmap showing the results of ssGSEA in 177 PDAC transcriptome datasets ranked according to the degree of immune cell infiltration. **D** Venn diagram showing the intersection of prognostic genes from FUSCC transcriptome sequencing and immune-related genes from TCGA database. **E** The flow chart showing our analysis process. **F** Univariate cox hazard analysis of these 327 genes in the data of TCGA and ICGC, and genes with P < 0.05 were shown in the table. **G** Venn diagram showed the intersection of two sets of data. **H** Kaplan-Meier survival curve of GFPT2 expression in tumor tissues from TCGA database was showed. **I** The expression of GFPT2 in tumor tissues and normal adjacent pancreas tissues from TCGA database was showed. **J** The correlation between the expression of GFPT2 and the infiltration of immune cells (B cells, CD4 + T cells, CD8 + T cells, neutrophils, macrophages and dendritic cells) was analyzed by Tumor Immune Estimation Resource (TIMER).

To identify immune-related prognostic genes in PDAC, we performed transcriptome sequencing in tumor samples from 22 patients diagnosed with PDAC from FUSCC. Of these 22 patients, 12 had an overall survival of less than 1 year, and 10 had an overall survival of more than 3 years (Fig. 1A). By comparing the transcriptome sequencing of patients in the two groups, we identified 1491 differentially expressed genes (Fig. 1B). We further conducted single-sample gene set enrichment analysis of 177 PDAC transcriptome datasets from The Cancer Genome Atlas (TCGA) and divided the patients into two groups with high or low levels of immune cell infiltration (Fig. 1C). A total of 967 differentially expressed genes were identified in the two groups (Fig. S1). In the two sets of data, 327 genes overlapped, which might be immune-related prognostic genes (Fig. 1D). To further verify these immune-related prognostic genes, we performed univariate Cox hazard analysis of these 327 genes in the TCGA and International Cancer Genome Consortium (ICGC) data (Fig. 1E), and used forest maps to show these genes with P value < 0.05 (Fig. 1F). We found that only GFPT2 was statistically significant in the hazard analysis of both TCGA and ICGC data (Fig. 1G). We found that GFPT2 was generally more highly expressed in tumor tissues and that the high expression of GFPT2 was associated with worse prognosis (Fig. 1H, I). In addition, we analyzed the correlation between the expression of GFPT2 and the infiltration of immune cells (B cells, CD4 + T cells, CD8 + T cells, neutrophils, macrophages and dendritic cells) and found that macrophages had the best correlation with GFPT2 (Fig. 1J). Therefore, we identified GFPT2 as an immune-related prognostic gene and further explored the effect of GFPT2 on macrophages.

### GFPT2 overexpressing pancreatic cancer cells promoted macrophage M2 polarization

**Fig. 2:**
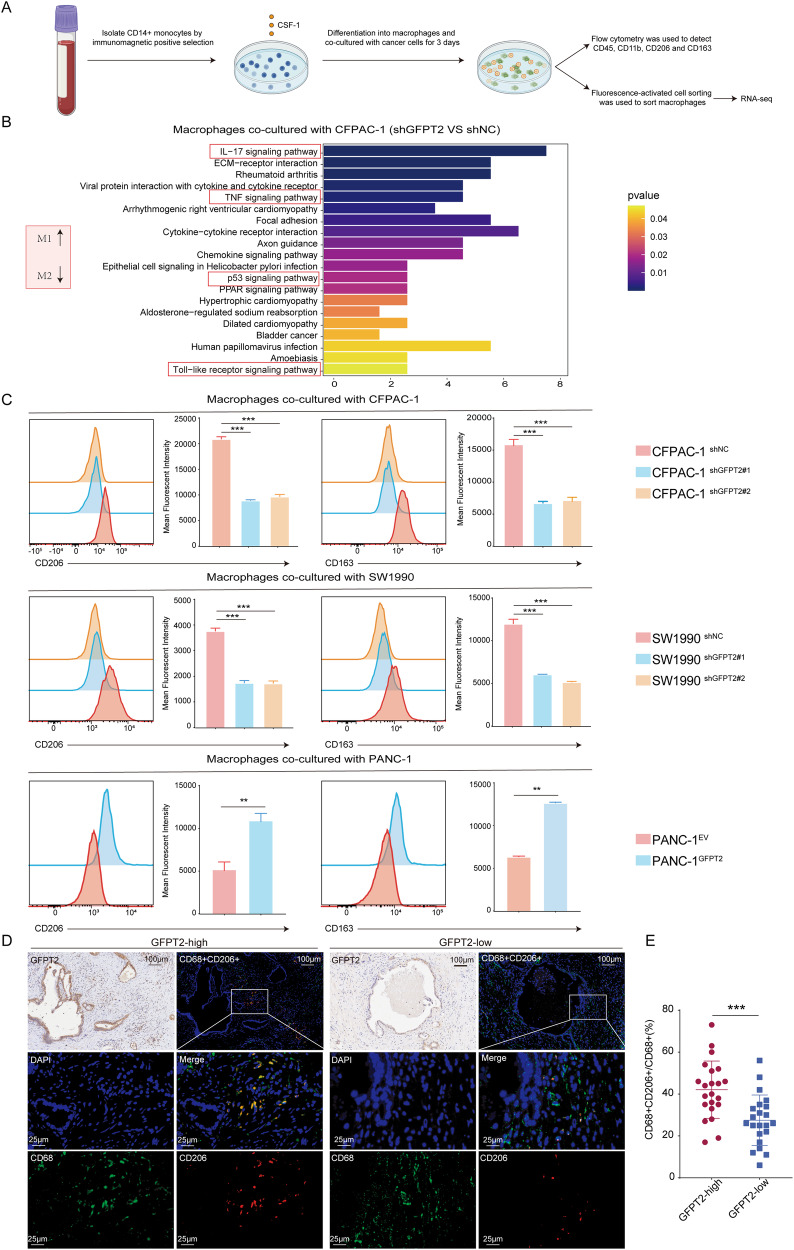
GFPT2 overexpressing pancreatic cancer cells promoted macrophage M2 polarization. **A** The pattern diagram showed the coculture system of pancreatic cancer cells and macrophages which was derived from CD14+ monocytes. **B** KEGG analysis of transcriptome sequencing data on macrophages cocultured with GFPT2-knockdown CFPAC-1 cells. **C** CFPAC-1, SW1990 and PANC-1 stable cell lines were cocultured with macrophages for 3 days and then M2 macrophage were analyzed by flow cytometry with CD11b, CD45, CD163 and CD206 antibodies. **D**, **E** Multiplex immunohistochemistry assay showing the macrophage M2 polarization by CD206 and CD68 antibodies and the proportion of M2 in all macrophages was calculated.

To further explore the impact of GFPT2 on macrophage polarization, we constructed an in vitro model of pancreatic cancer cells with stable knockdown or overexpression of GFPT2 co-cultured with macrophages. First, we detected the expression of GFPT2 in pancreatic cancer cell lines. Compared to those in normal pancreatic duct cells (H6c7), SW1990 and CFPAC-1 had higher GFPT2 expression, while PANC-1, Capan-1 and MIAPACA2 had lower GFPT2 expression, and Patu8988 did not show a significant change (Fig. S2A and S2B). We constructed GFPT2 knockdown cell lines in SW1990 and CFPAC-1 and GFPT2 overexpression cell lines in PANC-1 (Fig. S2C-S2E). Then, pancreatic cancer cells were cocultured with CD14+ monocytes, which can differentiate into macrophages (Fig. 2A) [13]. Next, we performed transcriptome sequencing on macrophages that cocultured with GFPT2-knockdown CFPAC-1 cells and further conducted Kyoto Encyclopedia of Genes and Genomes (KEGG) analysis. The results showed that after coculture, macrophages showed changes in pathways related to M1 macrophage activation or pathways related to inhibition of M2 macrophages, such as the IL-17 signaling pathway [14], TNF signaling pathway [15], p53 signaling pathway [16], and Toll-like receptor signaling pathway (Fig. 2B) [17], We found that the GFPT2 knockdown cell lines, SW1990 and CFPAC-1, reduced the proportion of M2 macrophages in the coculture system by flow cytometry analysis (Fig. 2C). The opposite trend was observed in the GFPT2 overexpressing PANC-1 cell line (Fig. 2C). In human PDAC tissues, through a multiplex immunohistochemistry, we used a CD68 antibody to detect macrophages, and both CD68 and CD206 positive cells represented M2 macrophages (Fig. 2D). By calculating the proportion of CD68 and CD206 double positive cells in all CD68 positive cells, we confirmed that the proportion of macrophage M2 polarization was higher in tissues with higher GFPT2 expression (Fig. 2E). These results indicated that GFPT2 overexpression in pancreatic cancer cells promoted macrophage M2 polarization.

### GFPT2 promoted the proliferation and migration of pancreatic cancer cells

**Fig. 3:**
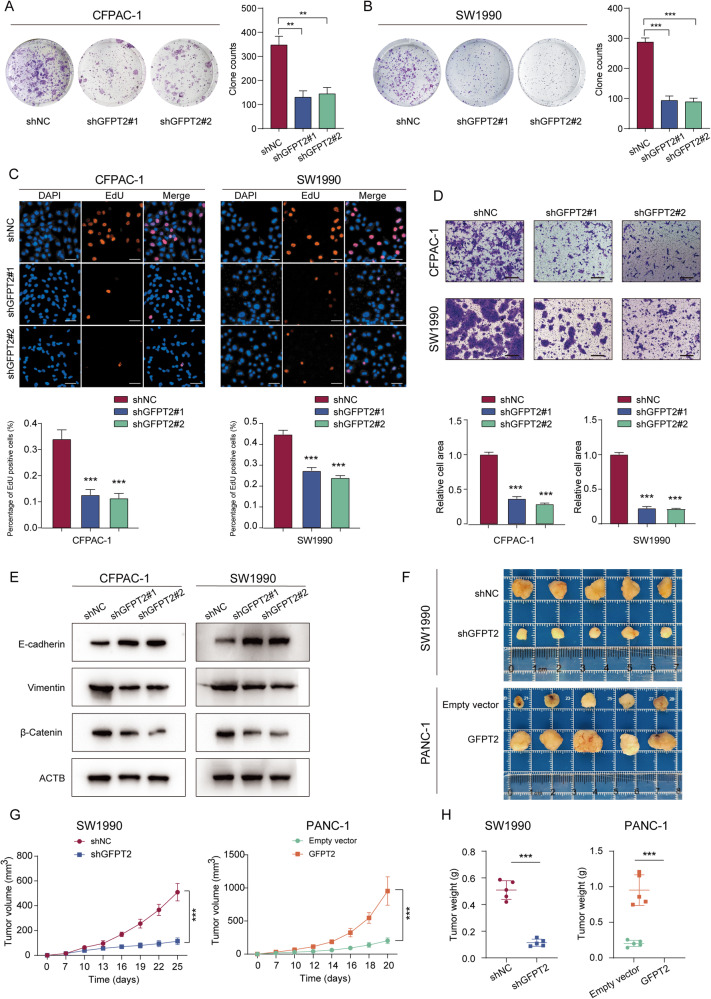
GFPT2 promoted the proliferation and migration of pancreatic cancer cells. **A**, **B** Colony formation assay was performed with CFPAC-1 and SW1990 stable cell lines. **C** 5-Ethynyl -2′- deoxy uridine (EdU) was used to detect the proliferation of CFPAC-1 and SW1990 stable cell lines. Scale bars, 50 μm. **D** Transwell assays were used to detect the migration abilities of CFPAC-1 and SW1990 stable cell lines. Scale bars, 200 μm. **E** The protein levels of E-cadherin, vimentin and β-catenin were measured by western blotting in CFPAC-1, SW1990 and PANC-1 stable cell lines. **F** The image displayed the subcutaneous implanted tumor of GFPT2-knockdown SW1990 and GFPT2-overexpressed PANC-1 cell lines in nude mice. **G** The effect of GFPT2-knockdown or -overexpression on the growth trends of subcutaneous implanted tumors were shown. **H** The weight of tumors was weighed.

In addition to affecting the immune microenvironment of pancreatic cancer, GFPT2 has also been reported to be related to the malignant biological behavior of cancer cells in a variety of other cancers including colon cancer, breast cancer and serous ovarian cancer [18–20]. However, there are few reports on the effect of GFPT2 on pancreatic cancer cells. As described above, we found GFPT2 was generally more highly expressed in PDAC tumor tissues and that the high expression of GFPT2 was associated with worse prognosis (Fig. 1H and 1I). To further validate the function of GFPT2 in PDAC, we investigated the effects of GFPT2 on the proliferation and migration of pancreatic cancer cells. The colony formation assay showed that GFPT2 knockdown significantly inhibited the formation of colony in SW1990 and CFPAC-1 cells (Fig. 3A, B), while GFPT2 overexpression significantly promoted the formation of colony in PANC-1 cells (Fig. S3D). In SW1990 and CFPAC-1 cells, knockdown of GFPT2 inhibited cell proliferation (Fig. 3C and S3A). Correspondingly, in PANC-1 cells, GFPT2 overexpression promoted cell proliferation (Fig. S3B and S3C). In addition, GFPT2 knockdown significantly inhibited cell migration and increased the protein level of E-cadherin and reduced the levels of vimentin and β-catenin, which are markers of epithelial-mesenchymal transition, in SW1990 and CFPAC-1 cells (Fig. 3D, E). The opposite trend was also observed in GFPT2-overexpressing PANC-1 cells (Fig. S3E and S3F). We subcutaneously implanted the GFPT2-knockdown SW1990 cell line or GFPT2-overexpressing PANC-1 cell line in nude mice. The results showed that GFPT2 knockdown significantly inhibited tumor growth in nude mice. GFPT2 overexpression significantly promoted tumor growth in nude mice (Fig. 3F–H). These results indicated that GFPT2 functioned as an oncogene and promoted the proliferation and migration of pancreatic cancer cells.

### GFPT2 promoted O-GlcNAcylation in pancreatic cancer

**Fig. 4:**
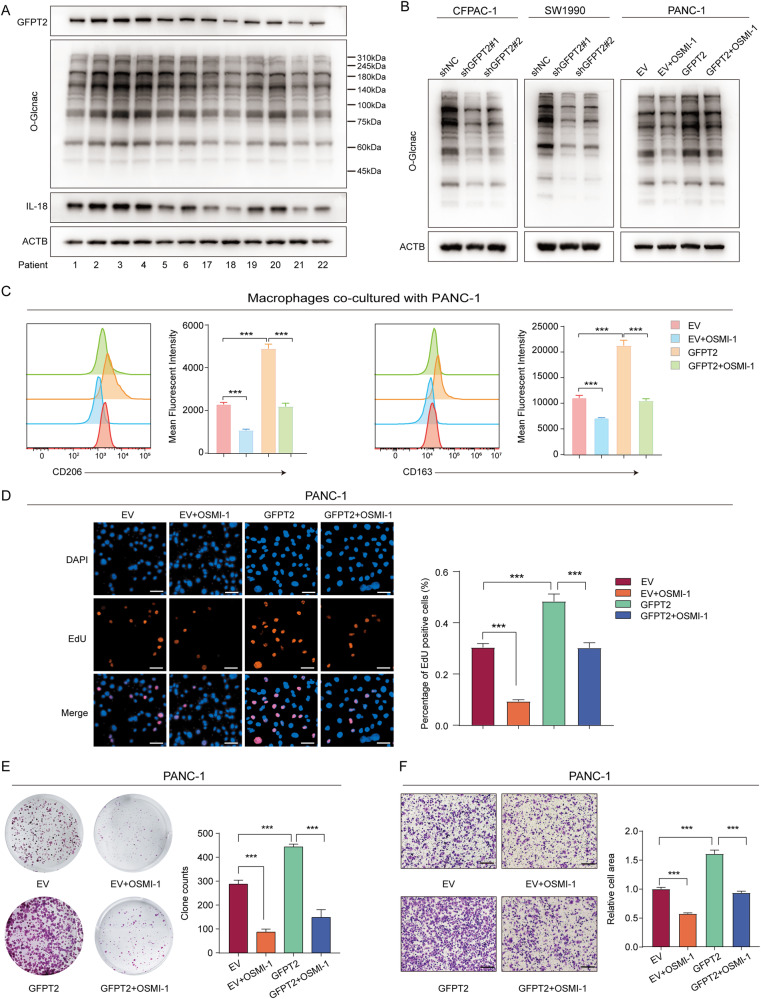
GFPT2 promoted O-GlcNAcylation in pancreatic cancer. **A** Western bolt analysis of GFPT2, IL-18 and overall levels of O-GlcNAcylation in 12 tumor tissues, which are derived from Fig. 1. **B** The overall level of O-GlcNAcylation was measured in pancreatic cancer cell lines (OSMI-1 (40 μm) for 48 h). **C** GFPT2 overexpressing PANC-1 cells were pretreated with OSMI-1 (40 μm) and then were cocultured with macrophages for 3 days and then M2 macrophage were analyzed by flow cytometry with CD45, CD11b, CD163 and CD206 antibodies. **D** EdU was used to detect the proliferation of GFPT2 overexpressing PANC-1 cells pretreated with OSMI-1 (40 μm). Scale bars, 50 μm. **E** Colony formation assay was performed with GFPT2 overexpressing PANC-1 cells pretreated with OSMI-1 (40 μm). **F** Transwell assays were used to detect the migration abilities of GFPT2 overexpressing PANC-1 cells pretreated with OSMI-1 (40 μm). Scale bars, 200 μm.

GFPT2 catalyzes the formation of glucosamine-6-phosphate from fructose-6-phosphate and glutamine and is one of the key enzymes in HBP, whose end product is UDP-GlcNAc [21]. UDP-GlcNAc is a substrate for O-GlcNAc transferase (OGT) to catalyze the attachment of O-GlcNAc moieties to proteins, which is defined as O-GlcNAcylation, a posttranslational modification that regulates fundamental cellular processes [22]. Therefore, it is reasonable to assume that GFPT2 promotes O-GlcNAcylation in PDAC. To confirm this assumption, we measured the overall levels of O-GlcNAcylation in tumor tissues and found that tumor tissues with high expression of GFPT2 tended to have high overall levels of O-GlcNAcylation (Fig. 4A). We further found that GFPT2 knockdown reduced the overall level of O-GlcNAcylation in SW1990 and CFPAC-1 cells (Fig. 4B). The opposite trend was also obtained in GFPT2 overexpressing PANC-1 cells (Fig. 4B). O-GlcNAcylation was reported to promote PDAC progression in a variety of ways [23–25]. Then, the role of O-GlcNAcylation in GFPT2-induced progression of PDAC was further validated. We found that the OGT inhibitor OSMI-1 could significantly reverse the overall O-GlcNAcylation caused by GFPT2 overexpression in PANC-1 cells (Fig. 4B). In addition, OSMI-1 significantly reversed the macrophage M2 polarization and malignant phenotype caused by GFPT2-overexpressing PANC-1 cells (Fig. 4C–F). These results indicated that GFPT2 overexpression in pancreatic cancer cells promoted macrophage M2 polarization and the malignant phenotype of PDAC by promoting O-GlcNAcylation.

### GFPT2 promoted the synthesis and secretion of IL-18 in pancreatic cancer

**Fig. 5:**
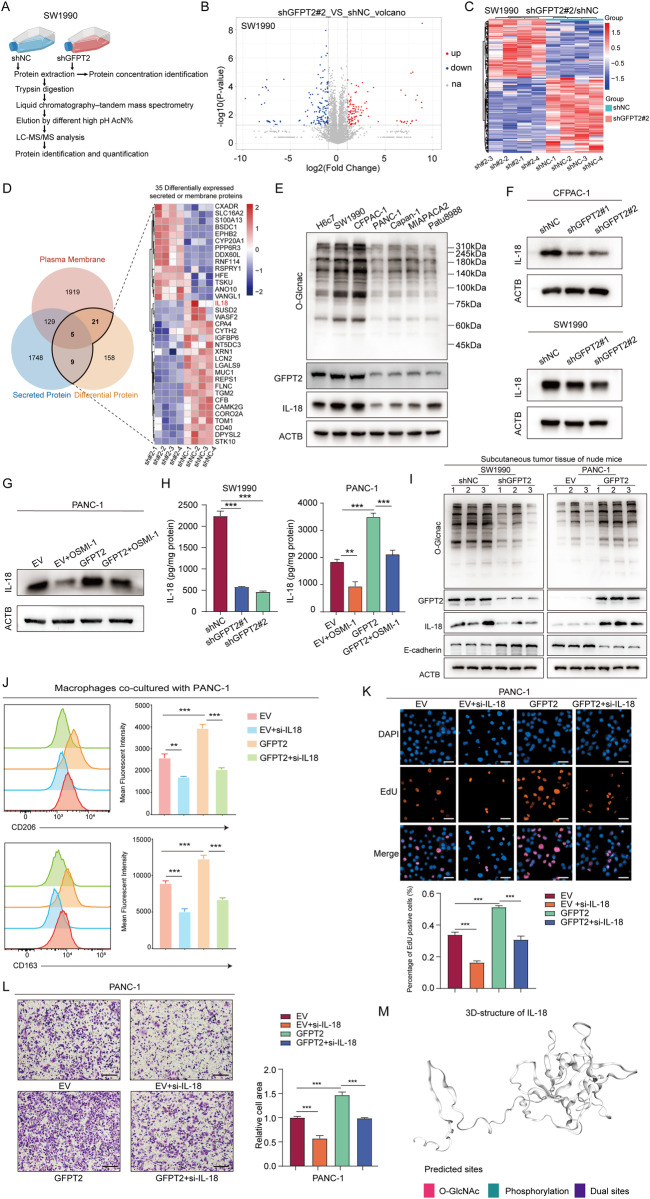
GFPT2 promoted the synthesis and secretion of IL-18 in pancreatic cancer. **A** The diagram shows the proteome analysis in GFPT2 knockdown and control SW1990 cells. **B**, **C** Volcano map and heatmap showing the changed proteins in the proteome analysis. **D** Venn diagram and heatmap showing the changed secreted proteins and membrane proteins in the proteome analysis. **E** Protein levels of IL-18 in pancreatic cell lines were measured by western blotting. **F** Protein levels of IL-18 in SW1990 and CFPAC-1 cells were measured by western blotting. **G** Protein levels of IL-18 in PANC-1 cells pretreated with OSMI-1 (40 μm) were detected by western blotting. **H** Secretion of IL-18 wad detected by an ELISA kit. **I** Protein levels of IL-18 in implanted tumors of nude were measured by western blotting. **J** Macrophages were cocultured with pancreatic cancer cells for 3 days and then M2 macrophage were analyzed by flow cytometry with CD45, CD11b, CD206 and CD163 antibodies. **K** EdU was used to detect the proliferation of GFPT2 overexpressing PANC-1 cells pretreated with siRNAs targeting IL-18. Scale bars, 50 μm. **L** Transwell assay was used to detect the migration abilities of GFPT2 overexpressing PANC-1 cells pretreated with siRNAs targeting IL-18. Scale bars, 200 μm. **M** The O-GlcNAcylation and phosphorylation site of IL-18 were predicted in the O-GlcNAc Database.

To explore the underlying molecular mechanism regulated by GFPT2 in PDAC, we performed a label-free quantitative proteomic strategy to screen proteins that are up- and downregulated by GFPT2 in SW1990 cells (Fig. 5A). Approximately 5800 proteins were identified and there were 110 GFPT2-downregulated and 144 GFPT2-upregulated proteins in our proteomic data (Fig. 5B, C). Considering that proteins generally considered to be reliable should contain two or more unique peptides, our focus shifted to the 193 proteins out of 254 differentially expressed proteins that met this requirement. The target protein should be able to affect both pancreatic cancer cells and macrophages. Therefore, we focused on secreted proteins, which could be secreted into the tumor environment to affect immune cells and tumor cells in return, and membrane proteins, which could mediate the communication between tumor cells and macrophages. By comparison with the secreted proteins and membrane proteins in the Human Protein Atlas (https://www.proteinatlas.org/), we found 35 up- or downregulated secreted proteins or membrane proteins in our proteomic data (Fig. 5D). Intriguingly, we found that IL-18 was significantly decreased in GFPT2 knockdown SW1990 cells (Fig. 5D). In our previous study, we confirmed that NF-κB-IL-18 signaling was activated and that secreted IL-18 promoted PDAC progression [26]. In addition, IL-18 receptor or Na-Cl cotransporter are expressed in macrophages in response to IL-18 stimulation [27]. IL-18 was also reported to act synergistically with IL-10 to amplify macrophage M2 polarization [28, 29]. In TCGA data, IL-18 was positively correlated with GFPT2 and predicted poor prognosis in PDAC (Fig. S4A and S4B). Therefore, we explored the role of IL-18 in GFPT2-mediated PDAC progression. The protein levels of IL-18 and GFPT2 in PDAC tissues and cell lines showed that IL-18 tended to change in line with GFPT2 and overall O-GlcNAcylation (Figs. 4A and 5E). Furthermore, GFPT2 knockdown inhibited the expression of IL-18 mRNA and protein as well as IL-18 secretion in SW1990 and CFPAC-1 cells, while the opposite trend was observed in GFPT2-overexpressing PANC-1 cells (Fig. 5F–H and S4C). More importantly, OSMI-1 significantly reversed IL-18 protein and secretion caused by GFPT2 overexpression in PANC-1 cells (Fig. 5G, H). In vivo experiments, in implanted tumors of nude mice, lower levels of protein O-GlcNAcylation and IL-18 were observed in GFPT2-knockdown cells, while the opposite trend was observed in GFPT2-overexpressing cells (Fig. 5I). Knockdown of IL-18 using siRNAs significantly reversed the macrophage M2 polarization and malignant phenotype caused by GFPT2 overexpression in PANC-1 cells (Fig. 5J–L). Protein O-GlcNAcylation is an important modification of protein. Thus, we further explored whether GFPT2 mediated the O-GlcNAcylation of IL-18. Indeed, through IP assay, we found that IL-18 did not be O-GlcNAcylated in pancreatic cancer cells (data not shown). Consistently, IL-18 was predicted to be an O-GlcNAcylation and phosphorylation double negative protein in the O-GlcNAc Database (https://www.oglcnac.mcw.edu/) (Fig. 5M). These results indicated that GFPT2 promoted macrophage M2 polarization and the malignant phenotype by promoting the synthesis and secretion of IL-18 in PDAC.

### GFPT2 promoted the O-GlcNAcylation and nuclear translocation of YBX1

**Fig. 6:**
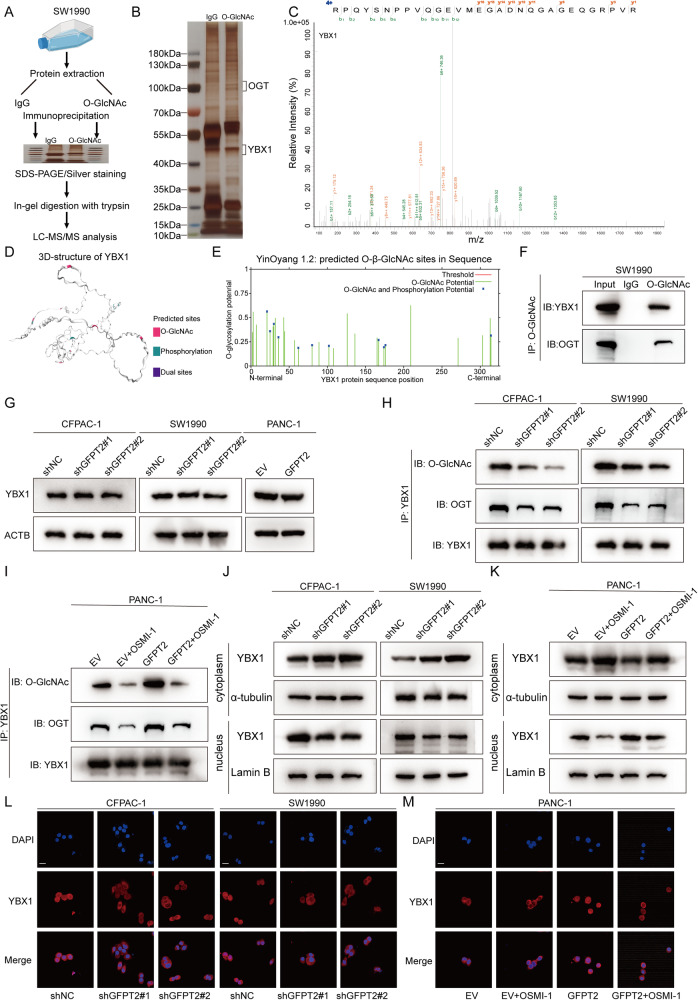
GFPT2 promoted the O-GlcNAcylation and nuclear translocation of YBX1. **A** The diagram showing the immunoprecipitation assay in SW1990 cells. **B** Silver staining of the proteins pulled down by IgG and O-GlcNAcylation antibodies. **C** Representative tandem MS spectrum of the RPQYSNPPVQGEVMEGADNQGAGEQGRPVR peptide from YBX1 as determined by IP-Mass Spec. **D** The O-GlcNAcylation and phosphorylation site of YBX1 were predicted in the O-GlcNAc Database. **E** The O-GlcNAcylation and phosphorylation site of YBX1 were predicted in YinOYang-1.2 database. **F** Immunoprecipitation was performed with IgG and O-GlcNAcylation antibodies and YBX1 and OGT proteins were detected by western blotting. **G** YBX1 was detected by western blotting in GFPT2-knockdown SW1990 and CFPAC-1 cells or GFPT2-overexpressing PANC-1 cells. **H** Immunoprecipitation was performed with a YBX1 antibody and YBX1, OGT and O-GlcNAcylation were detected by western blotting in CFPAC-1 and SW1990 cells with GFPT2 changes. **I** Immunoprecipitation was performed with YBX1 antibody and YBX1, OGT, and O-GlcNAcylation were detected by western blotting in PANC-1 cells with GFPT2-changed and OSMI-1 (40 μm) pretreated. **J** Western blotting to detect the change in cytoplasmic and nuclear YBX1 in SW1990 and CFPAC-1 cells with GFPT2 changes. **K** Western blotting to detected the change of cytoplasmic and nuclear YBX1 in PANC-1 cells with GFPT2 changes and OSMI-1 (40 μm) pretreated. **L** Immunofluorescence to detected the change of cytoplasmic and nuclear YBX1 in SW1990 and CFPAC-1 cells with GFPT2 changes. Scale bars, 20 μm. **M** Immunofluorescence to detected the change of cytoplasmic and nuclear YBX1 in PANC-1 cells with GFPT2 changes and OSMI-1 (40 μm) pretreated.

To explore the molecular mechanism by which GFPT2-mediated O-GlcNAcylation regulated IL-18 expression and secretion, we performed immunoprecipitation using an antibody against O-GlcNAcylation and further conducted mass spectrometry analysis (Fig. 6A). Compared to those pulled down by IgG, there were 221 proteins that were specifically pulled down by the O-GlcNAcylation antibody (Fig. 6B and Table S1). We noticed that Y-box binding protein-1 (YBX1) was specifically pulled down by the O-GlcNAcylation antibody (Fig. 6C and S5A-S5B). YBX1 was predicted to be an O-GlcNAcylated in the O-GlcNAc Database (https://www.oglcnac.mcw.edu/) and YinOYang-1.2 database (http://www.cbs.dtu.dk/services/YinOYang/) (Fig. 6D, E). We further performed coimmunoprecipitation to confirm that YBX1 and OGT could be pulled down by the O-GlcNAcylation antibody (Fig. 6F). YBX1 was reported to promote IL-4 expression in oral squamous cell carcinoma cells [30]. IL-4 is a canonical stimulant of macrophage M2 polarization [31, 32]. In addition, YBX1 functions as an oncogene in PDAC [33]. YBX1 also had the site to be O-GlcNAcylated [34]. Therefore, we speculated that YBX1 was an important downstream molecule in GFPT2-O-GlcNAcylation signaling. We found that the protein level of YBX1 was not changed in GFPT2 knockdown SW1990 and CFPAC-1 cells or GFPT2 overexpressing PANC-1 cells (Fig. 6G). O-GlcNAcylated YBX1 was significantly decreased when GFPT2 was knocked down or significantly increased when GFPT2 was overexpressed (Fig. 6H, I). In addition, YBX1 could bind to OGT, and the level of O-GlcNAcylated YBX1 was consistent with that of YBX1-bound OGT (Fig. 6H, I). The OGT inhibitor OSMI-1 significantly reversed the O-GlcNAcylation of YBX1 caused by GFPT2 overexpression in PANC-1 cells (Fig. 6I). Some O-GlcNAcylated proteins tended to translocate to the nucleus [35]. Indeed, nuclear localized YBX1 promoted cancer progression [36, 37]. We extracted cytoplasmic and nuclear proteins and found that nucleus-localized YBX1 was significantly reduced when GFPT2 was knocked down or significantly increased when GFPT2 was overexpressed (Fig. 6J and 6K). In addition, OSMI-1 reversed the YBX1 nuclear localization caused by GFPT2 overexpression (Fig. 6K). The effect of GFPT2 on YBX1 nuclear localization was further observed by immunofluorescence in SW1990 and CFPAC-1 cells with GFPT2 knockdown (Fig. 6L). The effect of OSMI-1 on GFPT2-induced YBX1 nuclear localization was also observed by immunofluorescence in PANC-1 cells (Fig. 6M). These results indicated that GFPT2 promoted the O-GlcNAcylation and nuclear translocation of YBX1 in PDAC.

### YBX1 nuclear localization promoted IL-18 transcription

**Fig. 7:**
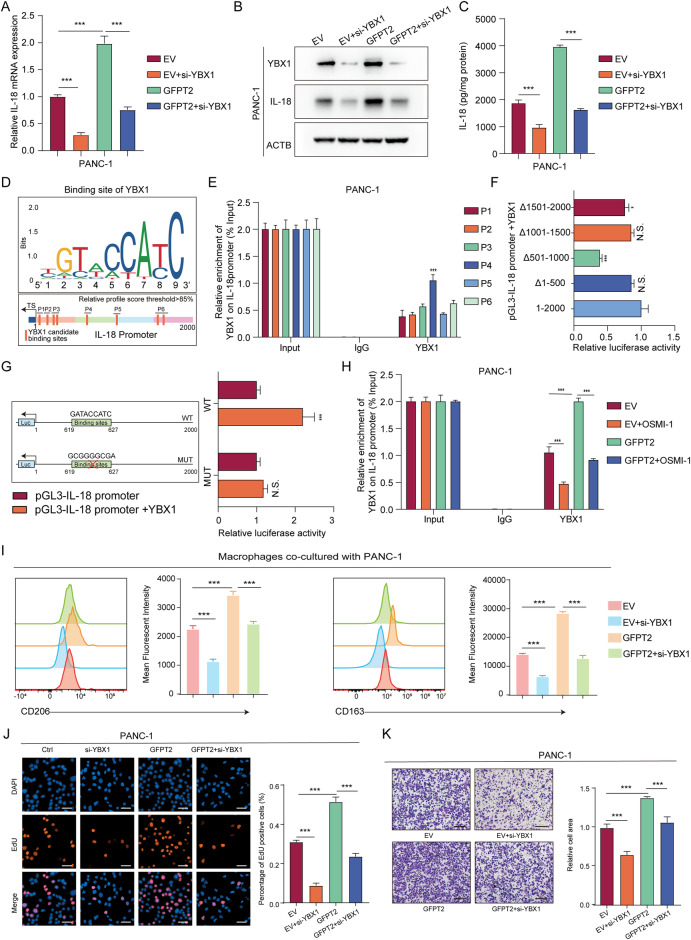
YBX1 nuclear localization promoted IL-18 transcription. **A** IL-18 mRNA was detected by qRT-PCR. **B** IL-18 protein was detected by western blotting. **C** The secretion of IL-18 was detected. **D** The binding site of YBX1 in the JASPAR database was showed and the binding sites in IL-18 promoter was showed. P1-6 showed 6 paired primers that covered the 8 binding sites in IL-18 promoter. **E** ChIP assay was performed with IgG and YBX1 antibodies in PANC-1 cells and qRT-PCR was performed using the designed 6 paired primers and the proportion of YBX1 binding sites was calculated. **F** Dual-luciferase reporter gene system was performed using full length or deleted ($\Delta$) promoters of IL-18 in 293 T cells. **G** Dual-luciferase reporter gene system was performed using the wild type site or the mutated site of YBX1 on IL-18 promoter in 293 T cells. **H** ChIP assay was performed with IgG and YBX1 antibodies in GFPT2-altered PANC-1 cells pretreated with OSMI-1 and qRT-PCR was performed using the primer 4 and the proportion of YBX1 binding sites were calculated. **I** Macrophage was cocultured with GFPT2 and YBX1-changed PANC-1 cells for 3 days and then M2 macrophage were analyzed by flow cytometry with CD11b, CD163 and CD206 antibodies. **J** EdU was used to detect the proliferation of GFPT2 overexpressing PANC-1 cells pretreated with siRNAs targeting YBX1. Scale bars, 50 μm. **K** Transwell assays were used to detect the migration abilities of GFPT2 overexpressing PANC-1 cells pretreated with siRNAs targeting YBX1. Scale bars, 200 μm.

To explore whether GFPT2 regulated IL-18 through YBX1. We used siRNAs to knock down YBX1 in PANC-1 cells, and found that YBX1 knockdown inhibited the expression and secretion of IL-18 and reversed the expression and secretion of IL-18 increased caused by GFPT2 overexpression (Fig. 7A–C). YBX1 is a DNA- and RNA-binding protein that regulates DNA repair, pre-mRNA transcription and splicing, mRNA packaging, and mRNA stability and translation [38, 39]. Thus, we speculated that YBX1 regulated the transcription or mRNA stability of IL-18. However, we found no significant change in IL-18 mRNA stability when YBX1was knocked down (Fig. S6). Therefore, YBX1 might function as a transcription factor to regulate IL-18 transcription. Indeed, nuclear localized YBX1 could function as a transcription factor. From the JASPAR database, we found that there were up to 8 binding sites of YBX1 in the promoter region of IL-18 (Fig. 7D). We further performed a ChIP assay using a YBX1 antibody. We designed 6 paired primers to cover these 8 binding sites in the IL-18 promoter and found that YBX1 mainly bound to promoter region of IL-18 at site covered by primer 4 (Fig. 7E). We performed dual-luciferase reporter gene system using full length (1–2000), 1–500 deleted ($\Delta$ 1–500), 501–1000 deleted ($\Delta$ 501–1000), 1001–1500 deleted ($\Delta$ 1001–1500), and 1501–2000 deleted ($\Delta$ 1501–2000) promoters of IL-18. The result showed that when the 501-1000 region of IL-18 promoter deleted, the luciferase activity significantly reduced (Fig. 7F). Coincidentally, the region corresponding to primer 4 was also between 501 and 1000. Thus, the site corresponding to primer 4 was the YBX1 binding site. We further performed dual-luciferase reporter gene system using the wild type site or the mutated site, and the luciferase activity was significantly increased after YBX1 overexpressing in the wild type group. However, in the mutated group, the luciferase activity did not show significantly change after YBX1 overexpressing (Fig. 7G). This indicated that YBX1 binds to the IL-18 promoter and promotes the transcription of the IL-18. We further explored the effect of GFPT2 and O-GlcNAcylation on the bonding of YBX1 on IL-18 promoter. Through ChIP assay using YBX1 antibody and followed qRT-PCR using primer 4, we found that overexpression of GFPT2 promoted the bonding of YBX1 on IL-18 promoter and OSMI-1 inhibited this bonding (Fig. 7H). In addition, knockdown of YBX1 significantly reversed the proliferation and migration of PANC-1 cells as well as macrophage M2 polarization caused by GFPT2 overexpression in PANC-1 cells (Fig. 7I–K). These results indicated that GFPT2 promoted the nuclear localization of YBX1 and then promoted IL-18 transcription.

### The GFPT2-YBX1-IL-18 signaling was further confirmed in clinical specimens and in vivo experiments

**Fig. 8:**
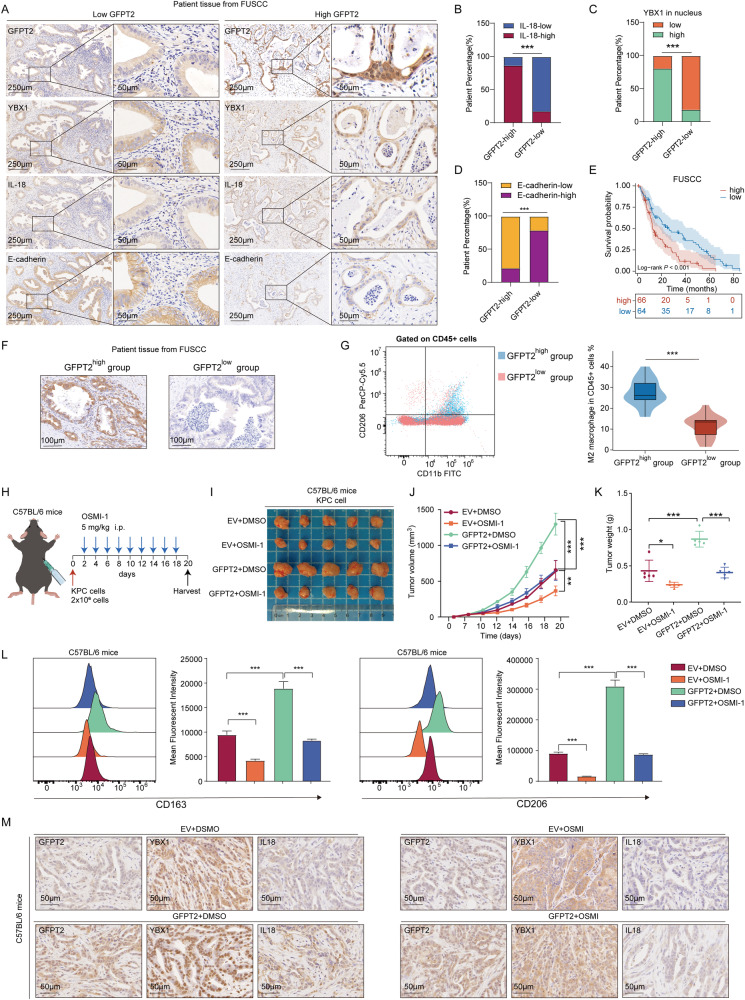
The GFPT2-YBX1-IL-18 signaling was further confirmed in clinical specimens and in vivo experiments. **A** Immunohistochemical detected GFPT2, YBX1, IL-18, and E-cadherin expression in human pancreatic cancer tissues. **B**–**D** Chi-square test was performed to analyzed the relationship of GFPT2 with YBX1, IL-18 or E-cadherin in human pancreatic cancer tissues. **E** Kaplan-Meier survival curve of GFPT2 expression in tumor tissues from our cohort was shown. **F** 40 cases of pancreatic cancer tissue samples were divided into high and low groups according to the expression level of GFPT2. **G** Flow cytometric analysis macrophages M2 polarization in the 40 fresh human pancreatic cancer tissues. **H**–**K** The subcutaneous implant tumor model of KPC cells in C57BL/6 mice. OSMI-1 or DMSO was intraperitoneally injected after cancer cells subcutaneously implanting. **L** Flow cytometric analysis macrophages M2 polarization in fresh subcutaneous implant tumors. **M** Immunohistochemical detected GFPT2, YBX1 and IL-18 expression in subcutaneous implant tumors of C57BL/6 mice.

In human PDAC tissues, though immunohistochemical assays, we also found that IL-18 and nuclear localized YBX1 more frequently occurred in tissues with higher GFPT2 expression, while E-cadherin more frequently occurred in tissues with lower GFPT2 expression (Fig. 8A–D). In addition, the patients with higher GFPT2 expression had a worse prognosis in our cohort (Fig. 8E). We prospectively collected surgical resection specimens from 40 patients with PDAC from FUSCC. After digestion and removal of red blood cells, flow cytometric analysis was performed on part of the fresh tissues to detect the degree of macrophages M2 polarization, and the other part was used to detect the GFPT2 expression level by immunohistochemistry. According to the level of GFPT2 expression, the cases were divided into GFPT2 ^high^ and GFPT2 ^low^ group (Fig. 8F). We found that patients with high expression of GFPT2 had a higher M2 polarization ratio (Fig. 8G). In subcutaneous tumors of nude mice, reduced IL-18 expression, protein O-GlcNAcylation and nuclear translocation of YBX1 were observed in GFPT2 knockdown cells, while the opposite trend was obtained in GFPT2 overexpressed cells (Fig. S7 and Fig. 5I). To explore whether targeting the GFPT2- O-GlcNAcylation signal axis has potential clinical significance, we used KPC cells to construct the subcutaneous implant tumor model in C57BL/6 mice, and OSIM-1 was injected intraperitoneally every other day (Fig. 8H). We found that OSMI-1 significantly inhibited tumor proliferation caused by GFPT2 (Fig. 8I–K). Flow cytometric analysis also showed that OSMI-1 significantly reserved macrophages M2 polarization caused by GFPT2 in the subcutaneous implant tumor model (Fig. 8L). In subcutaneous tumors of C57BL/6 mice, increased IL-18 expression and nuclear translocation of YBX1 were observed in GFPT2 overexpressing cells, while this effect can be reversed by OSMI-1 (Fig. 8M). These results suggested the existence of GFPT2-YBX-1-IL-18 signaling, and targeting this axis provided some insights into the combination therapy of PDAC.

## Discussion

PDAC will become the second leading cause of cancer-related mortality before 2040 [1]. Approximately 15–20% of patients have a chance of resectability when diagnosed, but only approximately 20% of these patients survive to 5 years. For locally advanced, unresectable, and metastatic disease, treatment is palliative [40]. The current standard of care for patients with advanced PDAC is a chemotherapy regimen based on gemcitabine. However, this treatment regimen only results in a modest benefit: an increase in survival of only 5 weeks [41]. There is an urgent need to find more effective treatment options.

Fortunately, immunotherapy has become a powerful clinical strategy for treating cancer. Currently, more than a dozen immunotherapies have been approved by the US Food and Drug Administration (FDA), including immune checkpoint inhibitors, cytokines for lymphocyte promotion, engineered T-cell therapies, vaccines, oncolytic viruses and bispecific antibodies [42]. In PDAC, due to the immunosuppressive tumor microenvironment, there is currently no immunotherapy approved by the FDA. However, combination immunotherapy strategies are currently being investigated and some strategies are ongoing in clinical trials [8]. This suggests that decoding the immune microenvironment of PDAC is of great significance.

In this study, we identified prognostic genes by transcriptome sequencing of clinical specimens in our center. We further explored the correlation between these identified prognostic genes and immune cell infiltration using TCGA data and then delineated immune-related prognostic genes in PDAC. Finally, we identified GFPT2 as an immune-related prognostic gene in PDAC. GFPT2 is the first and rate-limiting enzyme in the HBP. HBP is a glucose metabolism pathway leading to the generation of its end product uridine diphosphate N-acetylglucosamine, which is further utilized by OGT for protein modification, namely O-GlcNAcylation [43, 44]. Protein O-GlcNAcylation has been found to play a role in nearly all major cellular processes, including the cell cycle, genome maintenance, epigenetic regulation, protein synthesis/degradation, metabolic pathways, signaling pathways, stress response, and apoptosis [45]. Protein O-GlcNAcylation is also involved in cancer progression and the immune microenvironment. For instance, increased glucose uptake in M2-like tumor-associated macrophages fuels HBP for lysosomal OGT-mediated Cathepsin B O-GlcNAcylation to elevate its mature form and then promotes tumor metastasis and chemoresistance [46]. OGT-mediated O-GlcNAcylation of YTHDF2 enhanced its protein stability and oncogenic activity by inhibiting YTHDF2 ubiquitination and then promoted HBV-related hepatocellular carcinoma progression in an N6-methyladenosine-dependent manner [47]. In PDAC, high glucose enhanced HBP and O-GlcNAcylation of RRM1 to trigger nucleotide imbalance and KRAS mutation [24]. We observed a positive correlation between GFPT2 and total O-GlcNAcylation in the specimens and validated that GFPT2 promoted O-GlcNAcylation in pancreatic cancer cells. More importantly, GFPT2-mediated glycosylation was associated with macrophage M2 polarization and the malignant phenotype of PDAC. Thus, we searched for potential downstream targets by proteomics and focused on membrane proteins and secreted proteins, which are the main targets of O-GlcNAcylation and the main mediators of cell communication. We found that IL-18 was significantly decreased in GFPT2 knockdown SW1990 cells. We have confirmed that NF-κB-IL-18 signaling was activated and promoted PDAC progression [26]. In addition, IL-18 was reported to act synergistically with IL-10 to amplify macrophage M2 polarization [27, 28]. We further demonstrated that GFPT2-mediated O-GlcNAcylation promoted IL-18 secretion. Intriguingly, we found that IL-18 was transcriptionally regulated, but not protein O-GlcNAcylated. To explore the mechanism, we performed CO-IP assay to identify O-GlcNAcylated proteins. With the aid of mass spectrometry, we found that YBX1 was O-GlcNAcylated in pancreatic cancer cells. YBX1 was reported to be O-GlcNAcylated in hepatocellular carcinoma [28]. In addition, YBX1 promoted IL-4 expression in oral squamous cell carcinoma cells [30]. IL-4 is a canonical stimulant of macrophage M2 polarization [31, 32]. M2-type macrophages play an important role in the progression of PDAC and the immunosuppressive tumor microenvironment [48, 49]. Therefore, we speculated that YBX1 was the key mediator. Further experiments confirmed that O-GlcNAcylation of YBX1 resulted in nuclear translocation and then binding to the promoter of IL-18 to promote IL-18 transcription. This signaling pathway was further validated in specimens of PDAC.

In summary, we identified GFPT2 as an immune-related prognostic gene in PDAC and GFPT2-mediated O-GlcNAcylation promoted the O-GlcNAcylation and nuclear translocation of YBX1. Nuclear localized YBX1 functioned as a transcription factor to promote IL-18 transcription and secretion. Eventually, IL-18 promoted macrophage M2 polarization and the malignant phenotype of PDAC. Thus, our study might provide some insights into the combination therapy of HBP vulnerability and immunotherapy in PDAC.

### Supplementary information

Supplementary Figures and legends

Supplementary table

aj-checklist

Original Data File

## Data Availability

Data supporting the present study are available from the corresponding author upon reasonable request.
