# Supplementary material for: The GFPT2-O-GlcNAcylation-YBX1 axis promotes IL-18 secretion to regulate the tumor immune microenvironment in pancreatic cancer
Source: Cell Death Dis. 2024 Apr 4;15(4):244. doi: 10.1038/s41419-024-06589-7 (PMC10995196; doi:10.1038/s41419-024-06589-7)
Supplement: Supplementary file 1 — Supplementary Figures and legends [file 41419_2024_6589_MOESM1_ESM.docx]

**Supplementary Information**

**The GFPT2****-****O-GlcNAcylation-YBX1 axis** **promotes IL-18 secretion to regulate the** **tumor immune microenvironment in** **pancreatic cancer**

Supplementary Figures 1-7 and legends

**
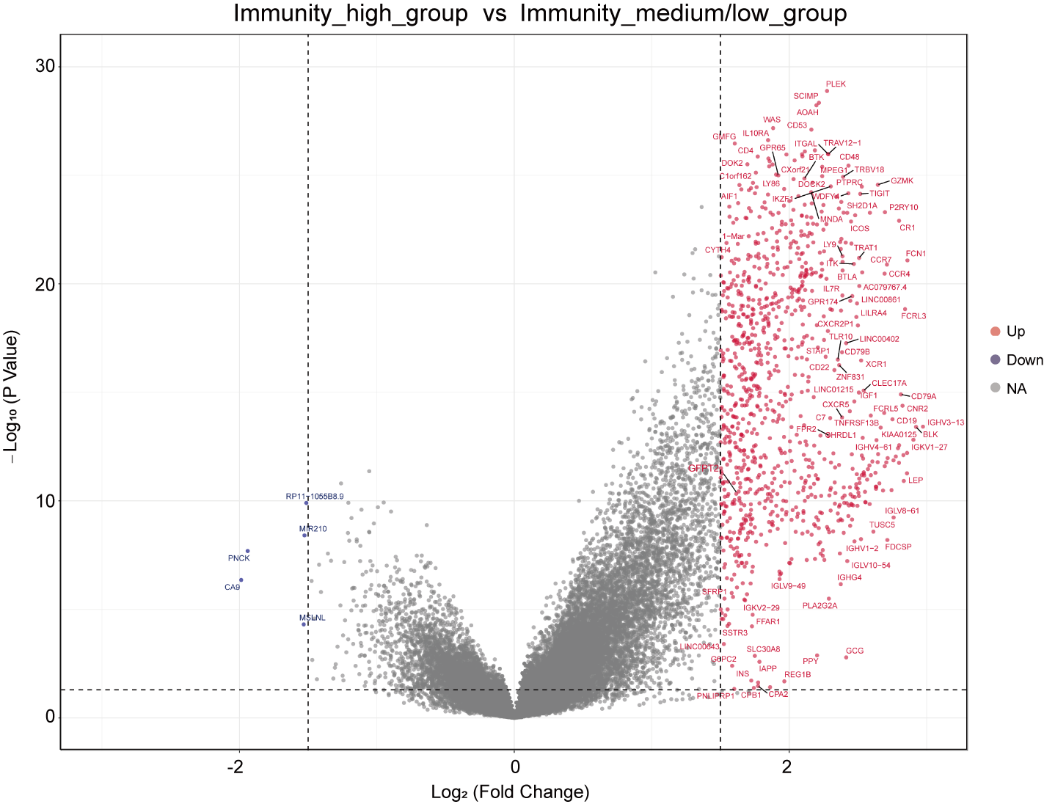
**

**Supplementary Figure 1. GFPT2 was an immune-related prognostic gene in pancreatic cancer.** Volcano map showed differences in gene expression between groups with high or low levels of immune cell infiltration (log_2_(Fold change) >1.5 or < -1.5).


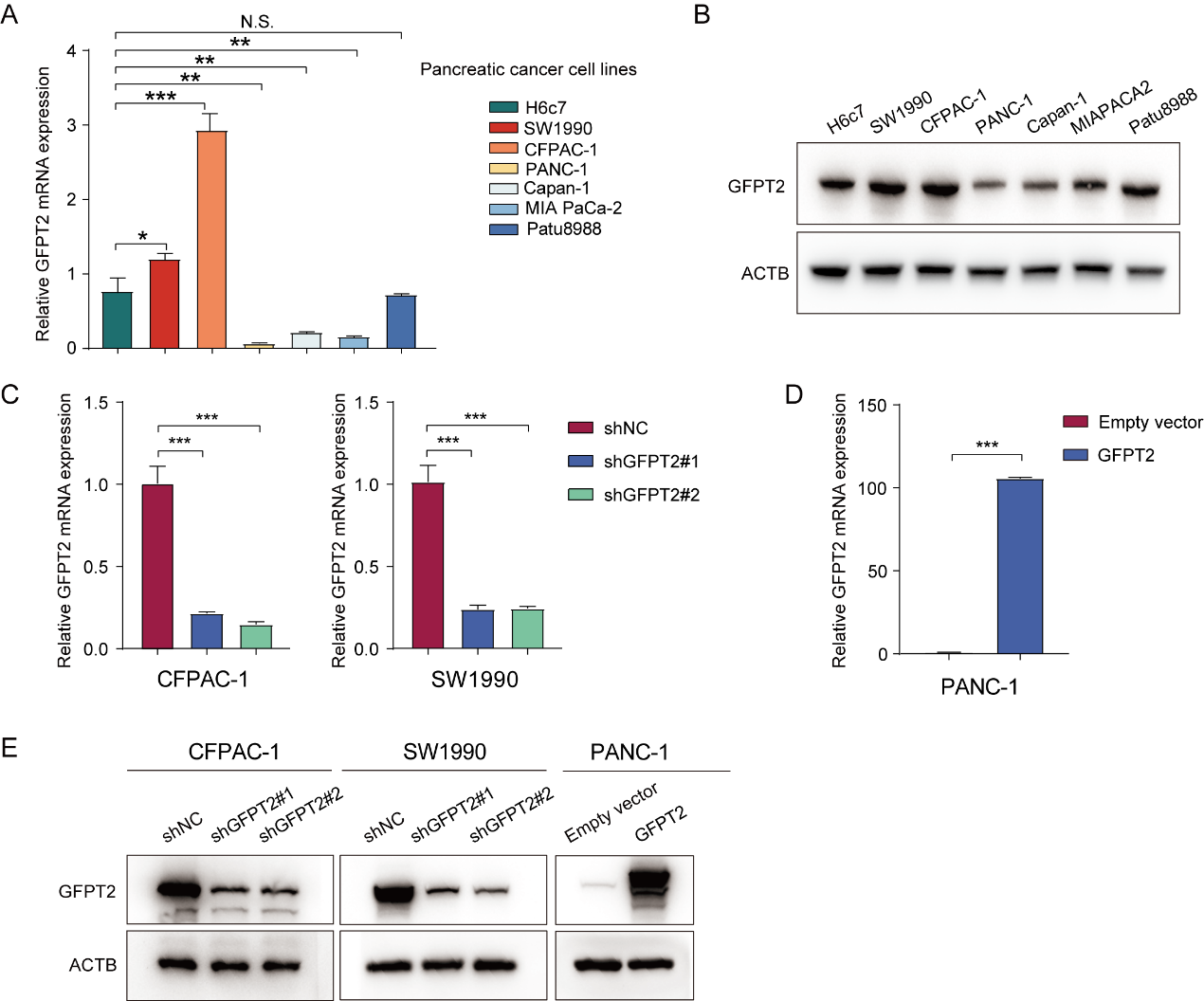


**Supplementary Figure 2.** **GFPT2 overexpressing pancreatic cancer cells promoted macrophage M2 polarization.** (A) GFPT2 mRNA of pancreatic cancer cell lines and H6c7 were detected by qRT-PCR. (B) GFPT2 protein levels of pancreatic cancer cell lines and H6c7 was measured by western blotting. (C-D) GFPT2 mRNA level was detected by qRT-PCR in SW1990, CFPAC-1 and PANC-1 stable cell lines. (E) GFPT2 protein level was measured by western blotting in SW1990, CFPAC-1 and PANC-1 stable cell lines.


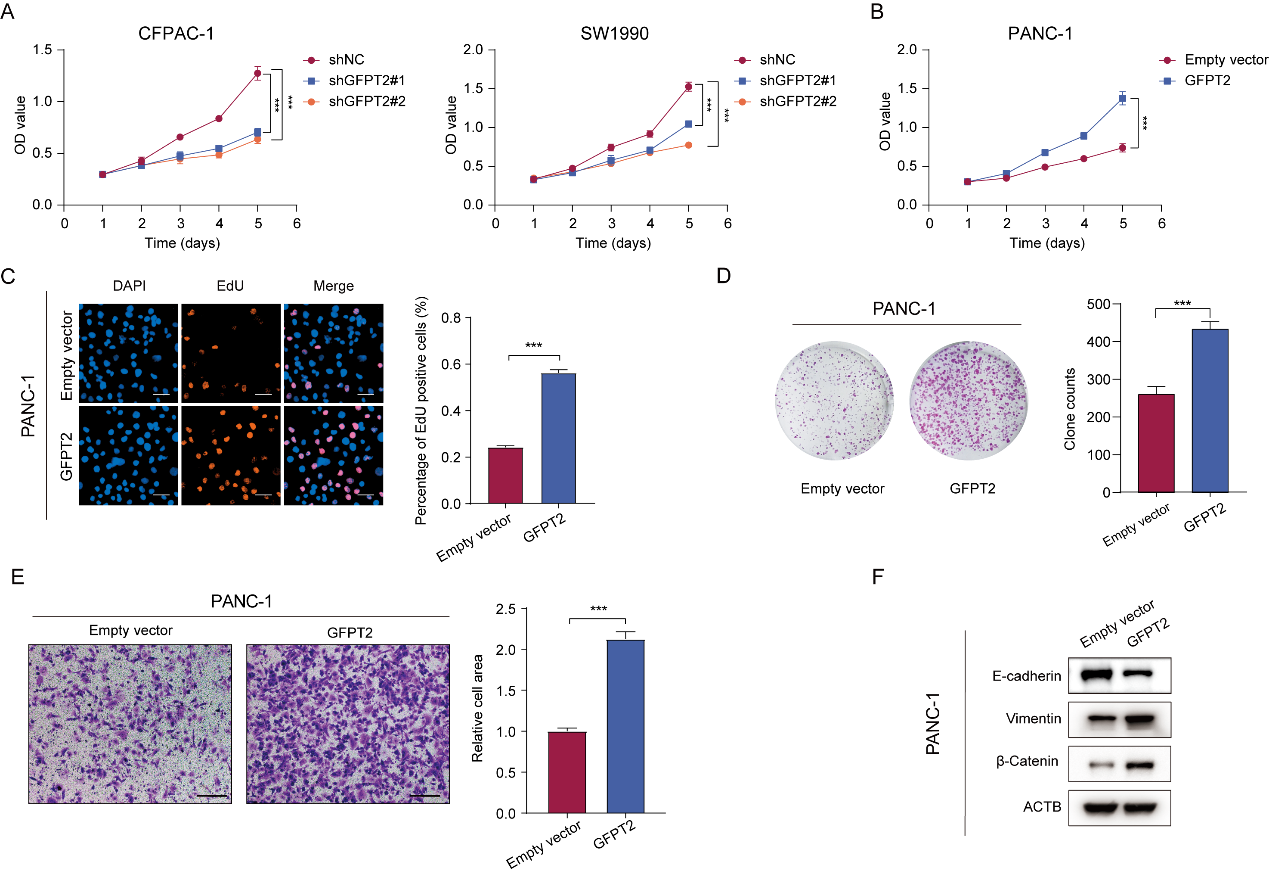


**Supplementary Figure 3.** **GFPT2 promoted the proliferation and migration of pancreatic cancer cells.** (A-B) CCK-8 assay was used to detect the proliferation of SW1990, CFPAC-1 and PANC-1 stable cell lines. (C) 5-Ethynyl -2’- deoxy uridine (EdU) was used to detect the proliferation of PANC-1 stable cell lines. Scale bars, 50 μm. (D) Colony formation assay was performed with PANC-1 stable cell lines. (E) Transwell assay was used to detect the migration abilities of PANC-1 stable cell lines. Scale bars, 200 μm. (F) The protein levels of E-cadherin, vimentin and β-catenin were detected by western blotting in SW1990 and CFPAC-1 stable cell lines.


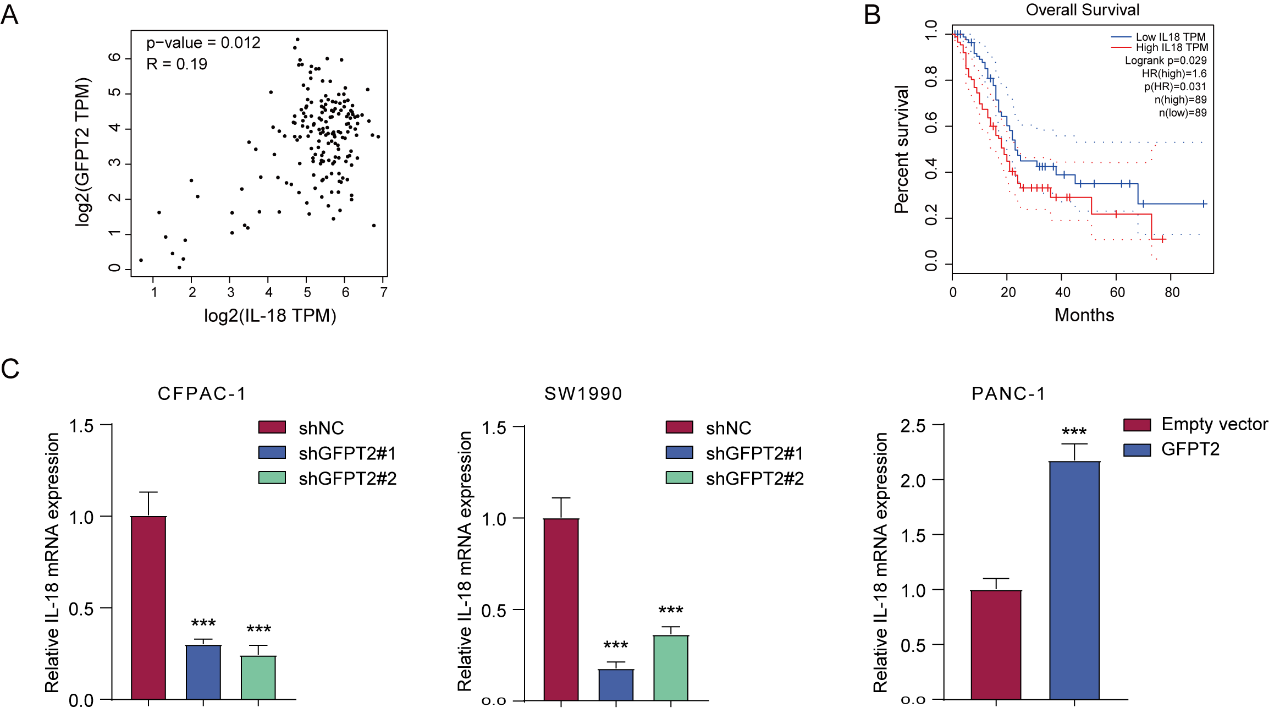


**Supplementary Figure 4. GFPT2 promoted the synthesis and secretion of IL-18 in pancreatic cancer.** (A) The correlation between GFPT2 and IL-18 in TCGA data was shown. (B) Kaplan-Meier survival curve of IL-18 expression in PDAC tumor tissues from TCGA database were shown. (C) mRNA levels of IL-18 were detected by qRT-PCR.


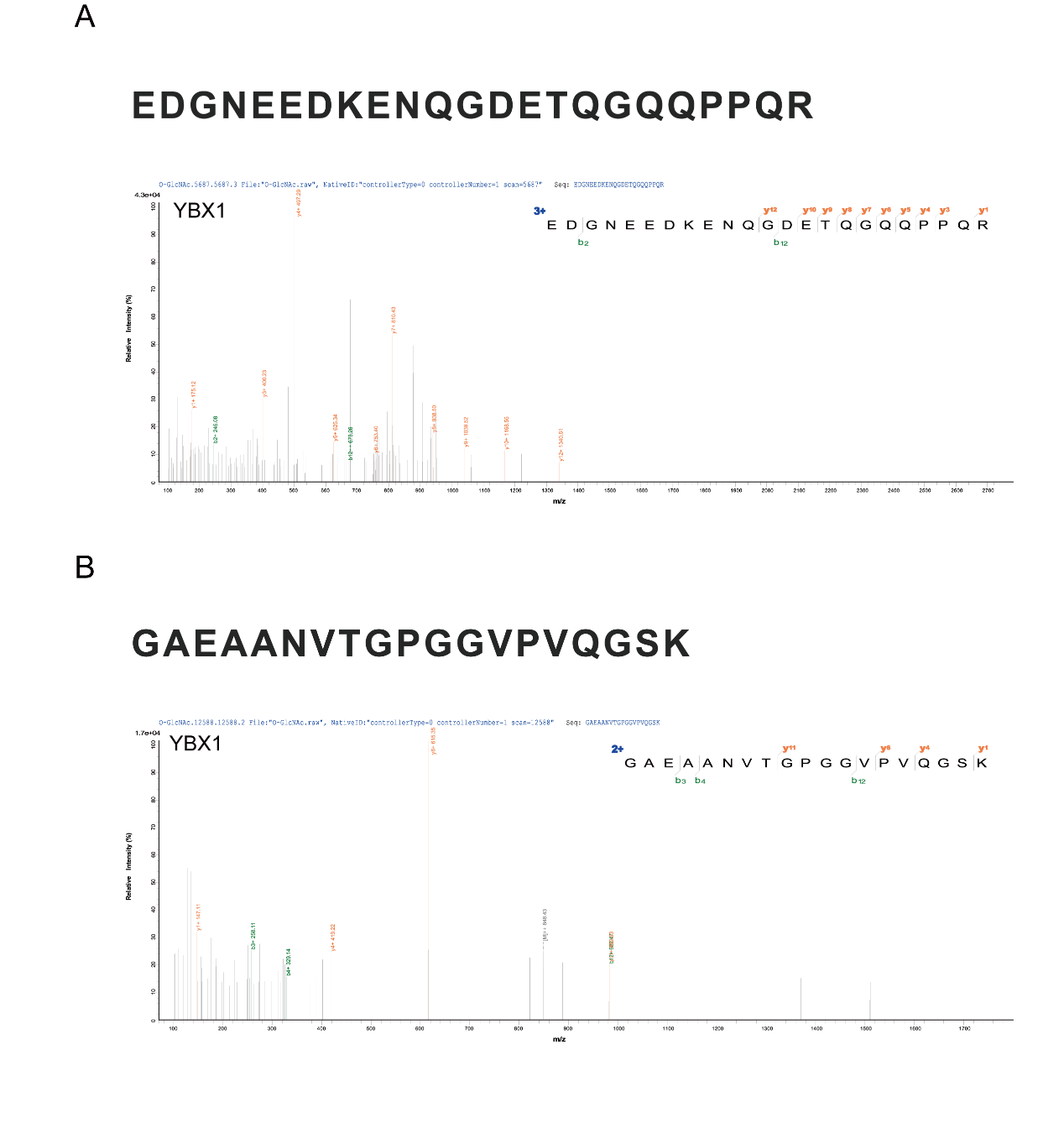


**Supplementary Figure 5. GFPT2 promoted the O-GlcNAcylation and nuclear translocation of YBX1.** (A) Representative tandem MS spectrum of the EDGNEEDKENQGDETQGQQPPQR peptide from YBX1 as determined by IP-Mass Spec. (B) Representative tandem MS spectrum of the GAEAANVTGPGGVPVQGSK peptide from YBX1 as determined by IP-Mass Spec.


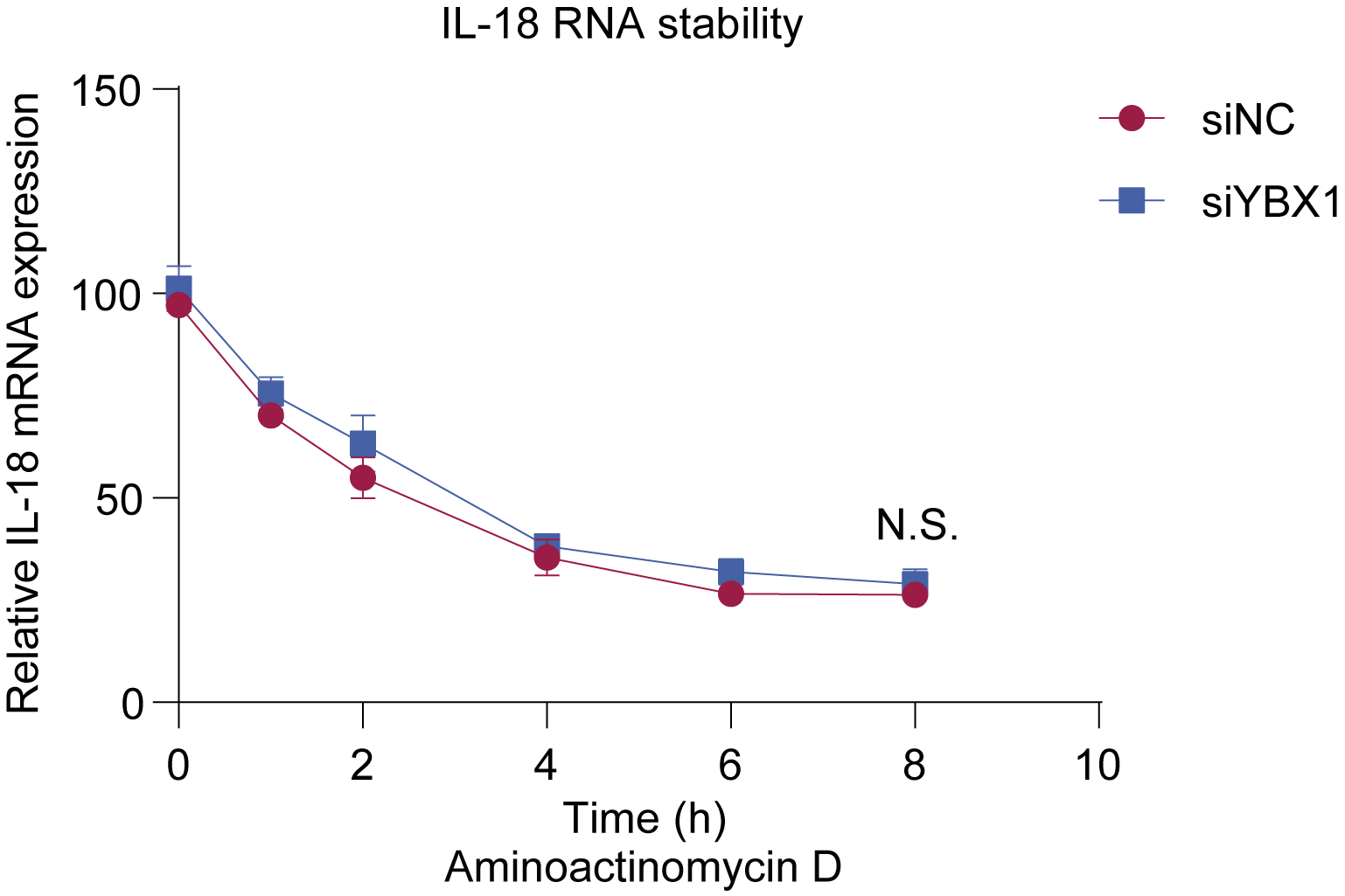


**Supplementary Figure 6. YBX1 nuclear localization promoted IL-18 transcription.** SW1990 cells were pretreated with aminoactinomycin D for the indicated time and then qRT-PCR was performed to detect IL-18 mRNA changes.


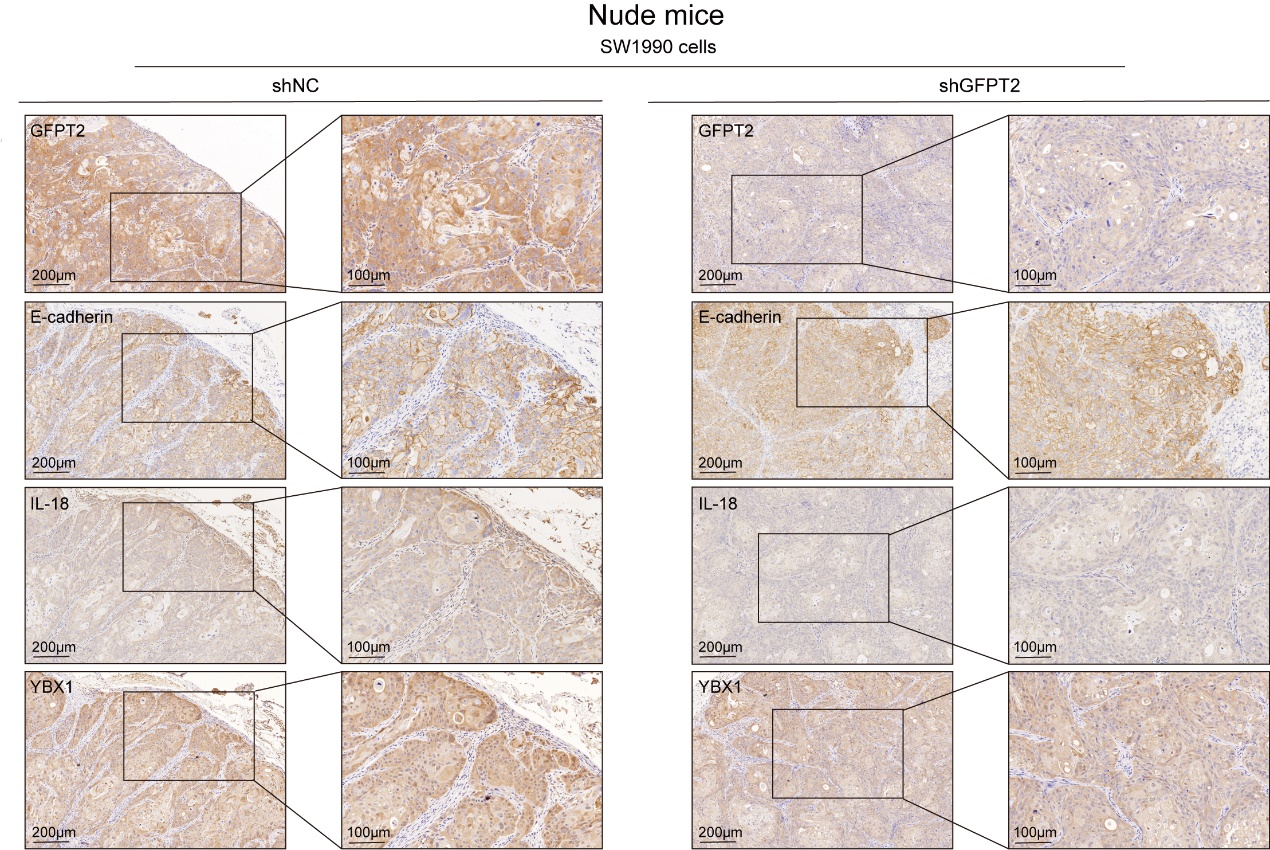


**Supplementary Figure 7. The GFPT2-YBX1-IL-18 signaling was further confirmed in vivo experiments.** Immunohistochemical assay to detect the expression of GFPT2, YBX1, IL-18 and E-cadherin in subcutaneously implanted tumor tissues of nude mice.
