# Supplementary material for: The GFPT2-O-GlcNAcylation-YBX1 axis promotes IL-18 secretion to regulate the tumor immune microenvironment in pancreatic cancer
Source: Cell Death Dis. 2024 Apr 4;15(4):244. doi: 10.1038/s41419-024-06589-7 (PMC10995196; doi:10.1038/s41419-024-06589-7)

Figure 3E

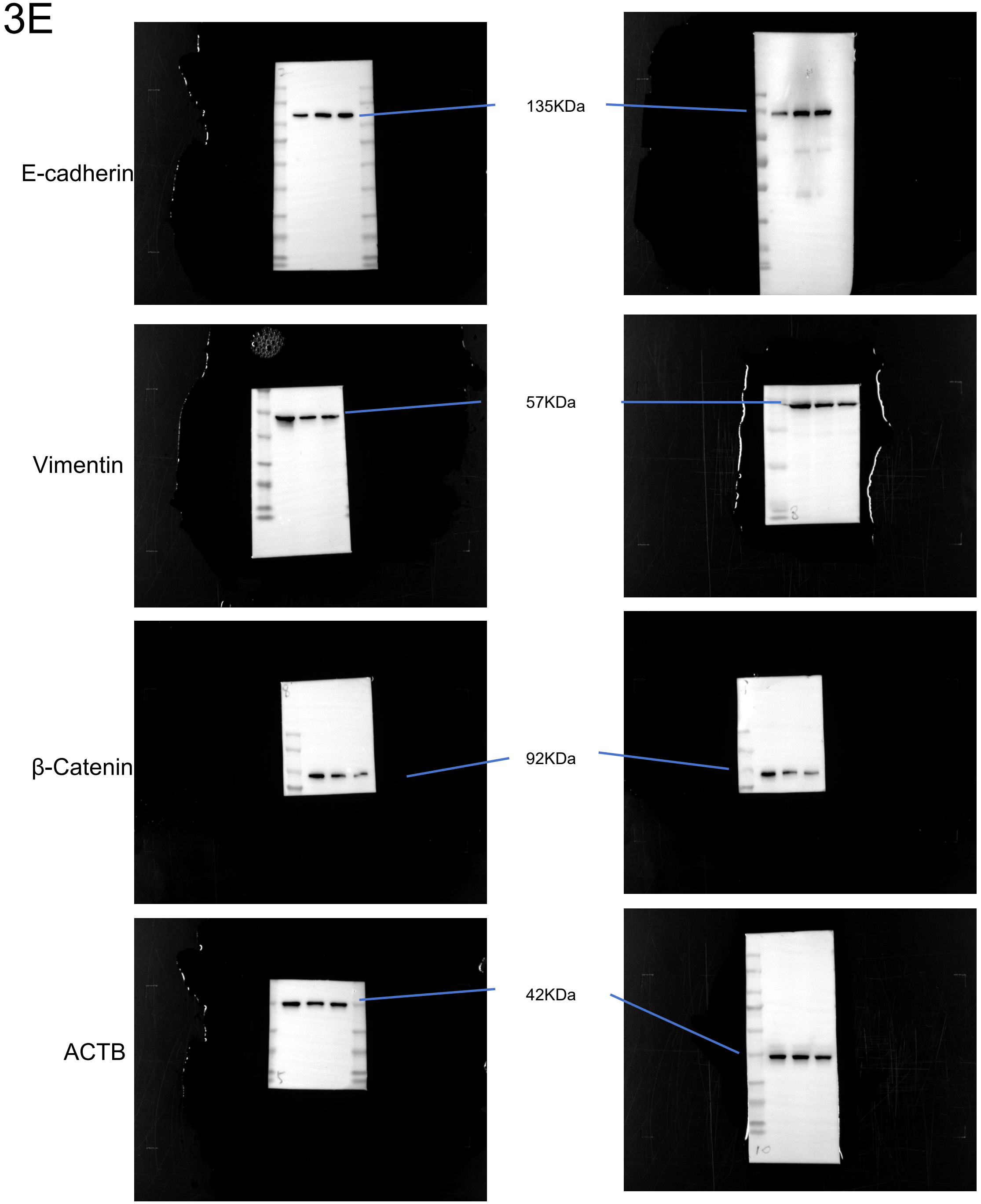
E-cadherin
135KDa
Vimentin
57KDa
β-Catenin
92KDa
ACTB
42KDa

Figure 4A

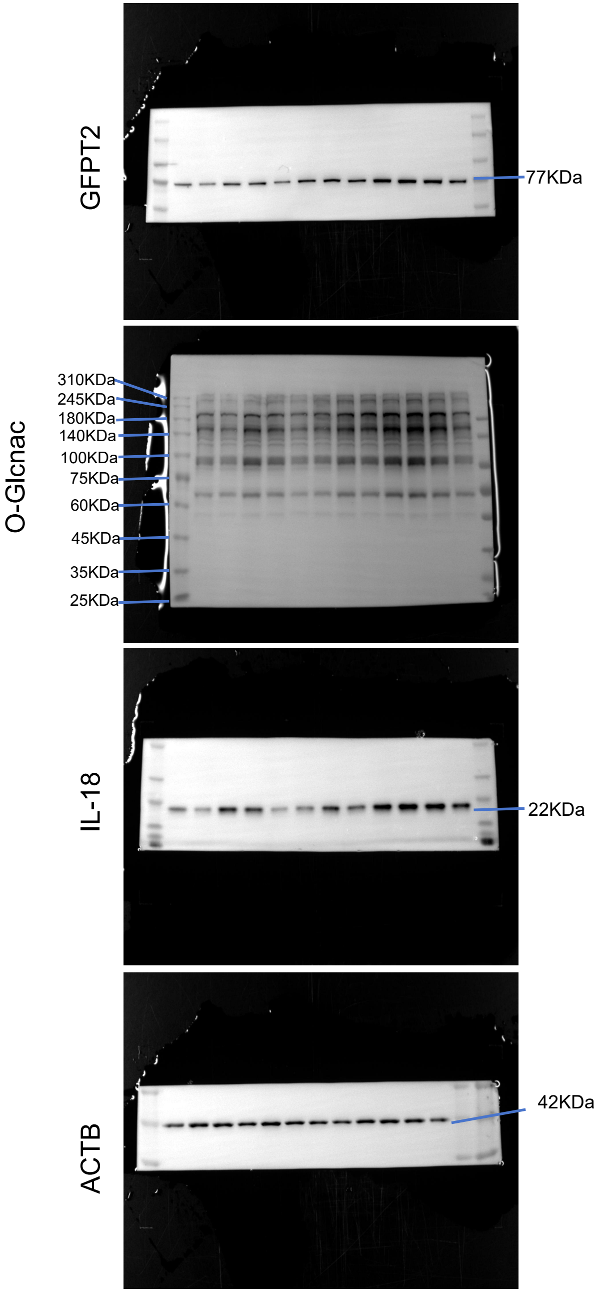

Figure 4B

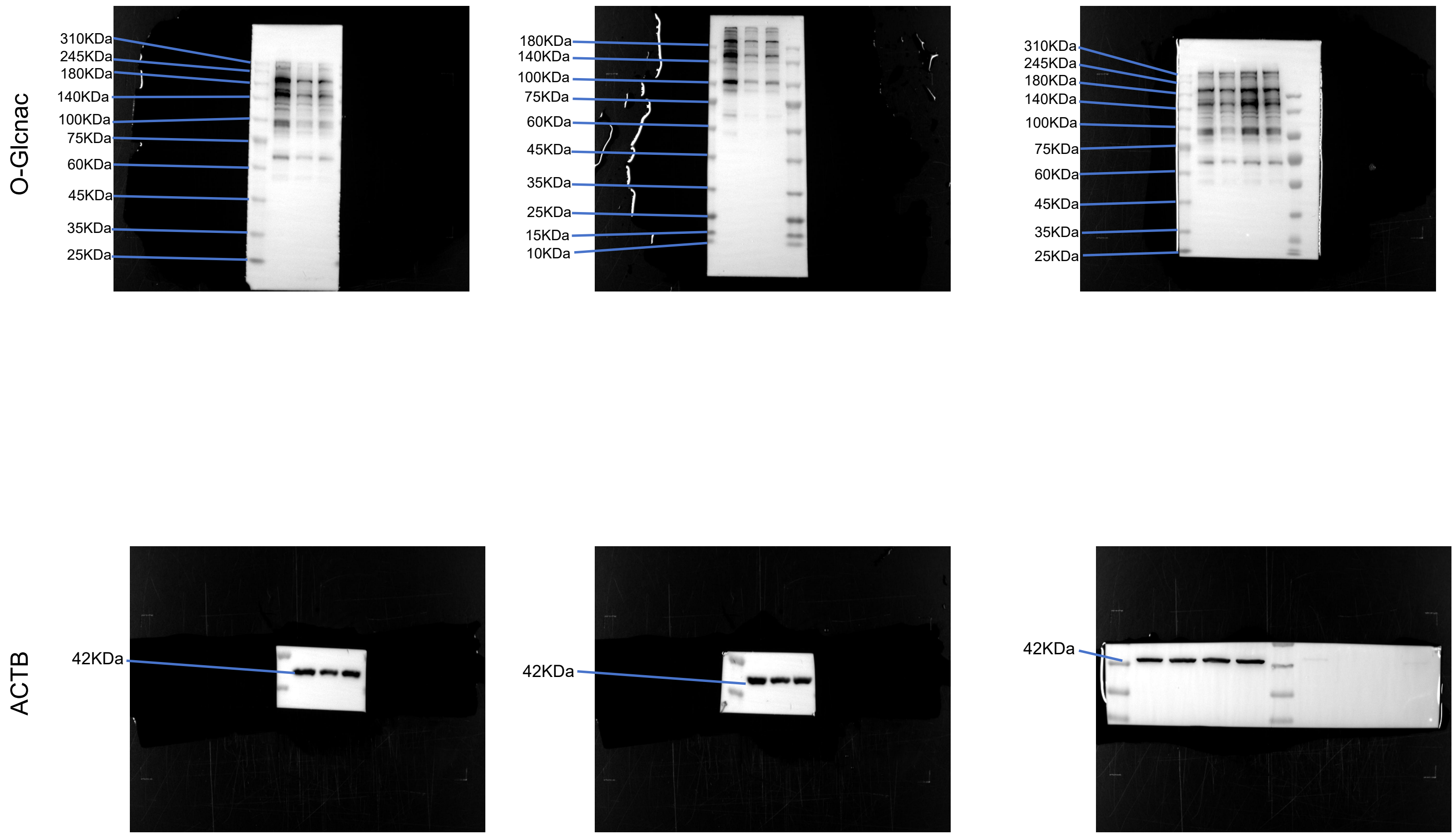

Figure 5E

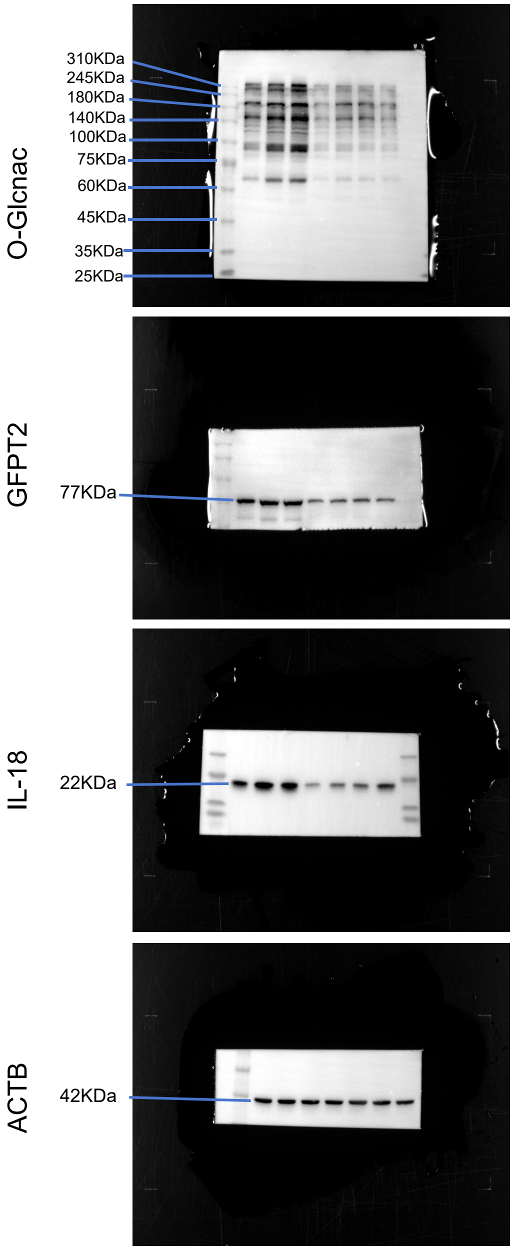

Figure 5F

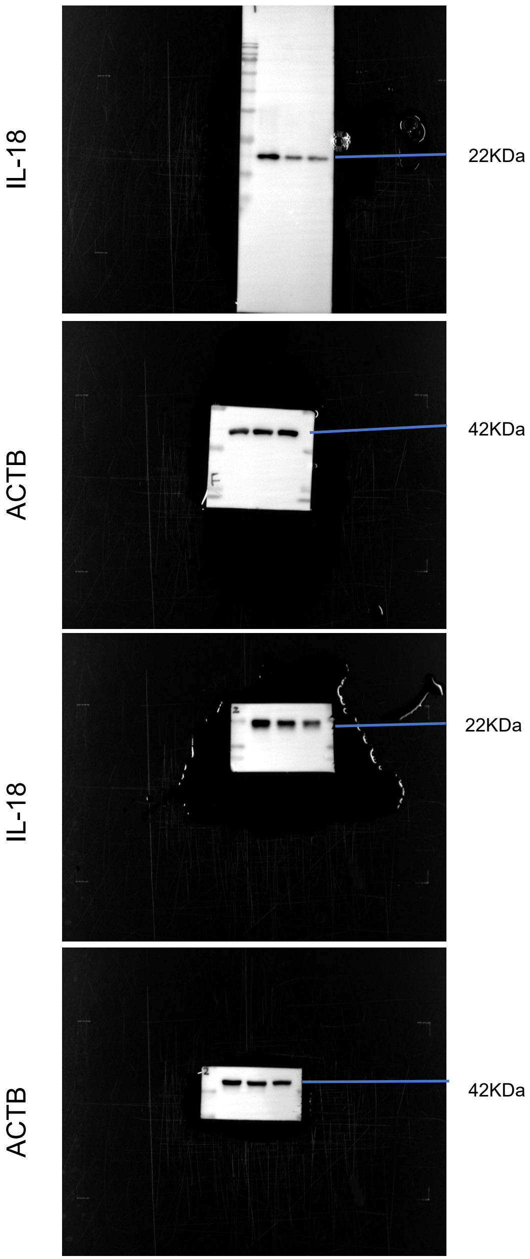

Figure 5G

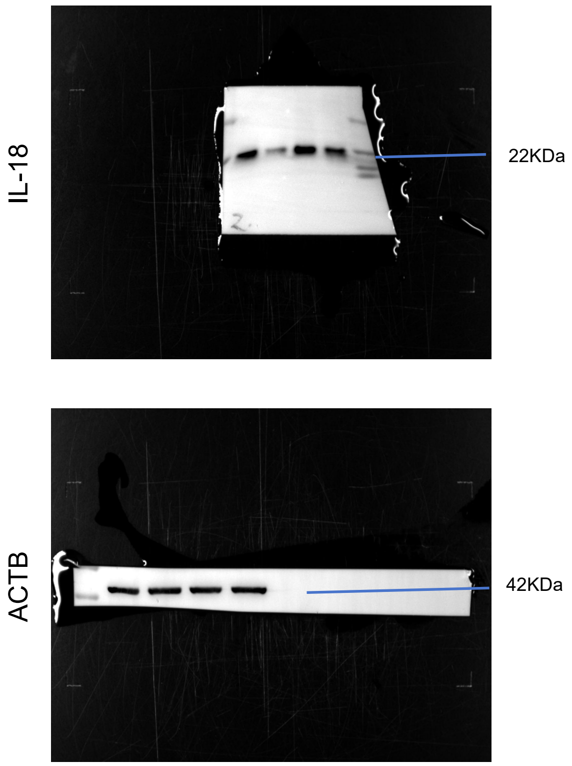

Figure 5I

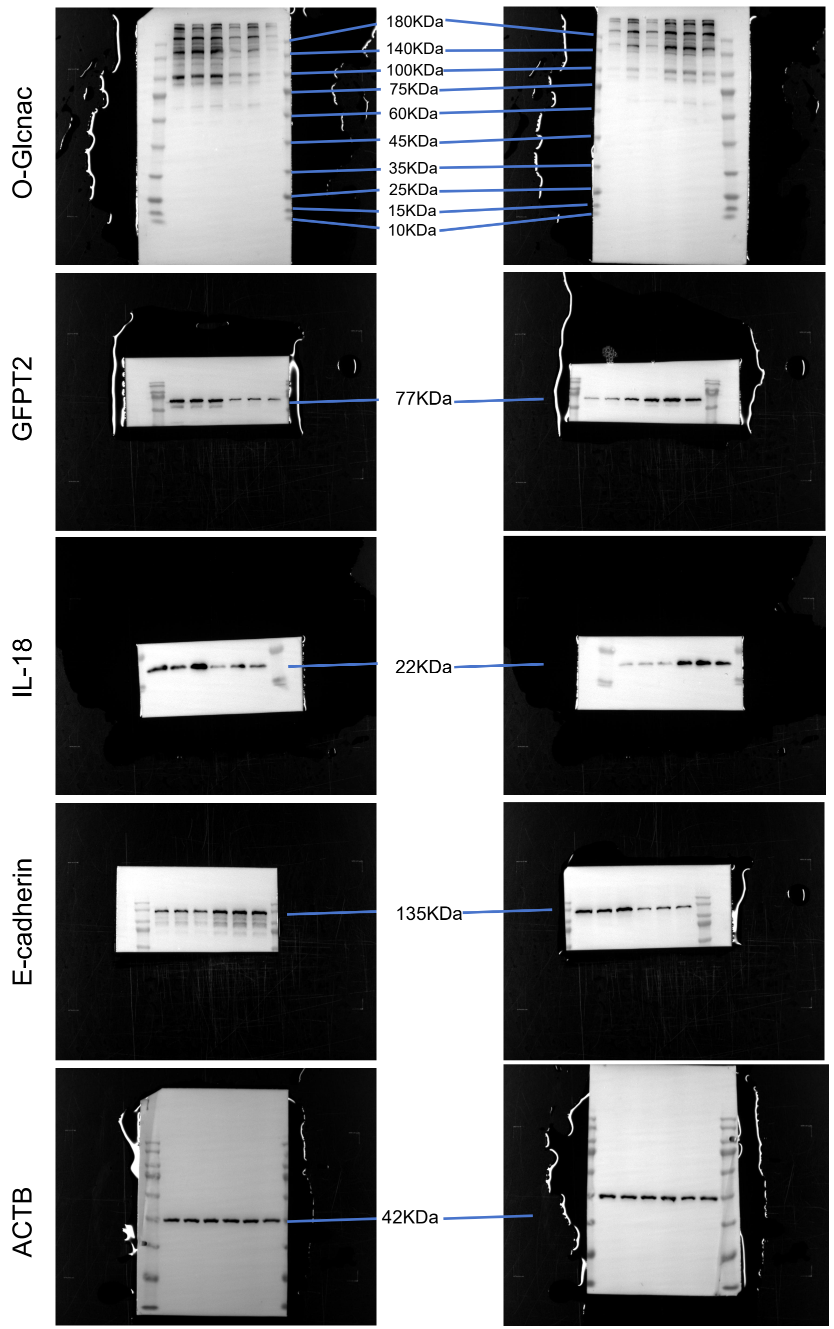
O-Glcnac
180KDa
140KDa
100KDa
75KDa
60KDa
45KDa
35KDa
25KDa
15KDa
10KDa
GFPT2
77KDa
IL-18
22KDa
E-cadherin
135KDa
ACTB
42KDa

Figure 6F

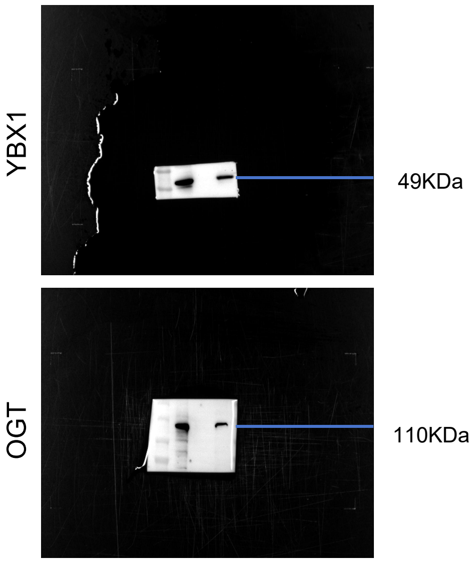

Figure 6G

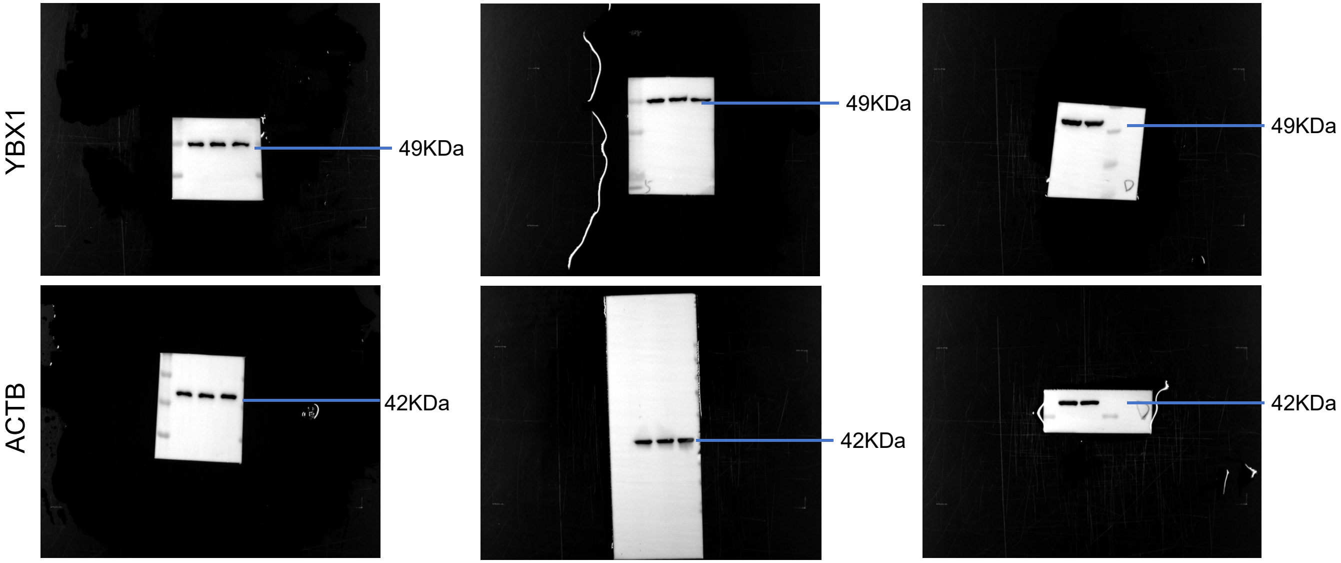

Figure 6H

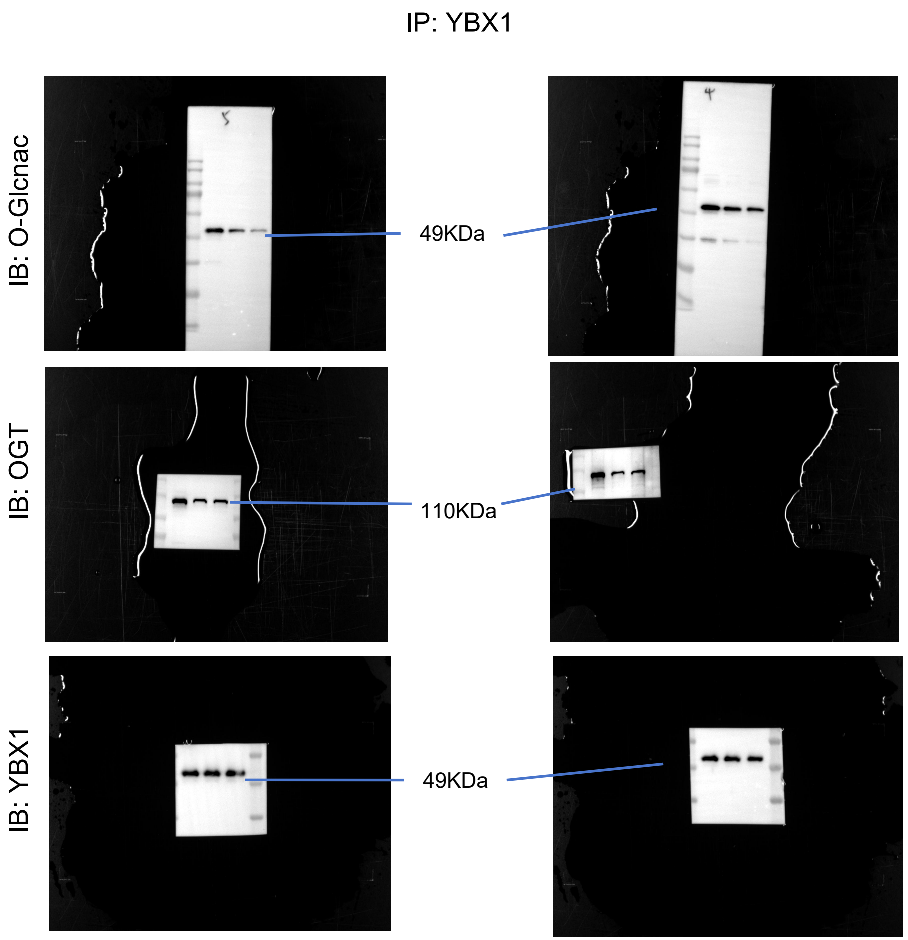

Figure 6I

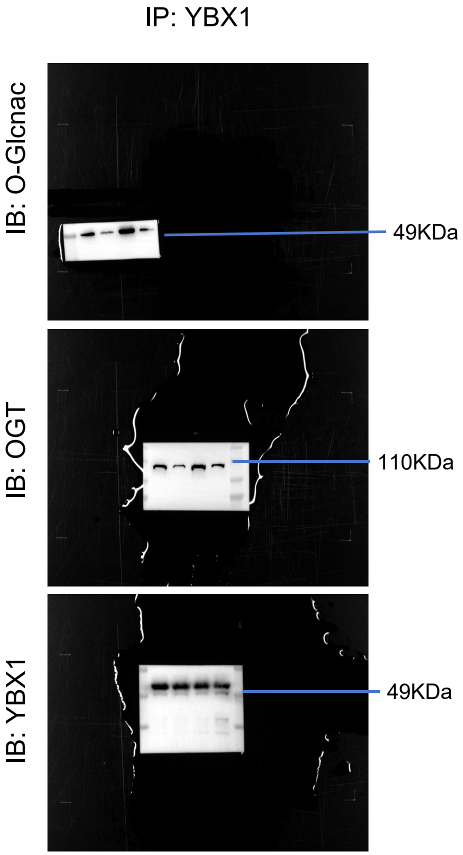

Figure 6J

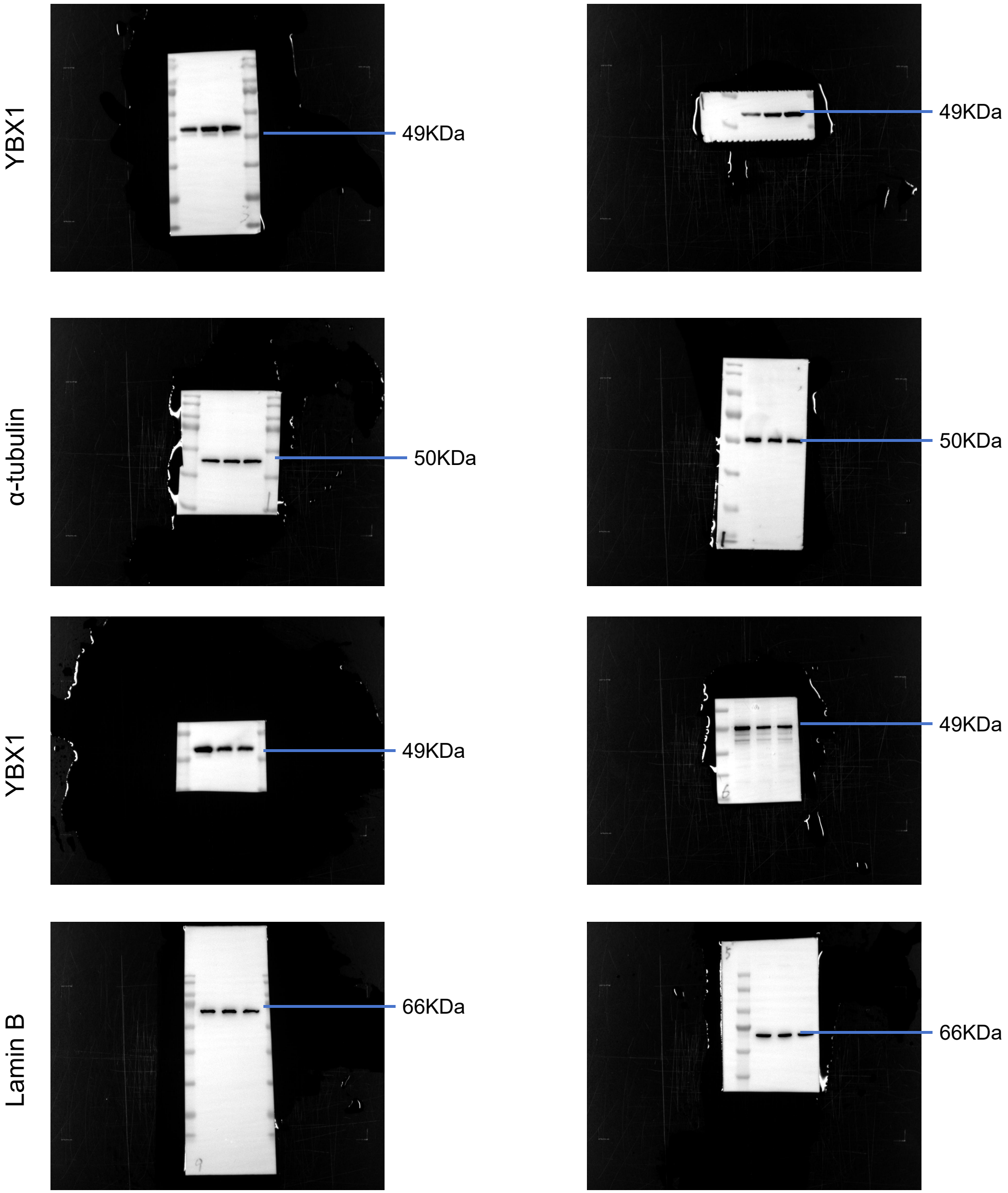

Figure 6K

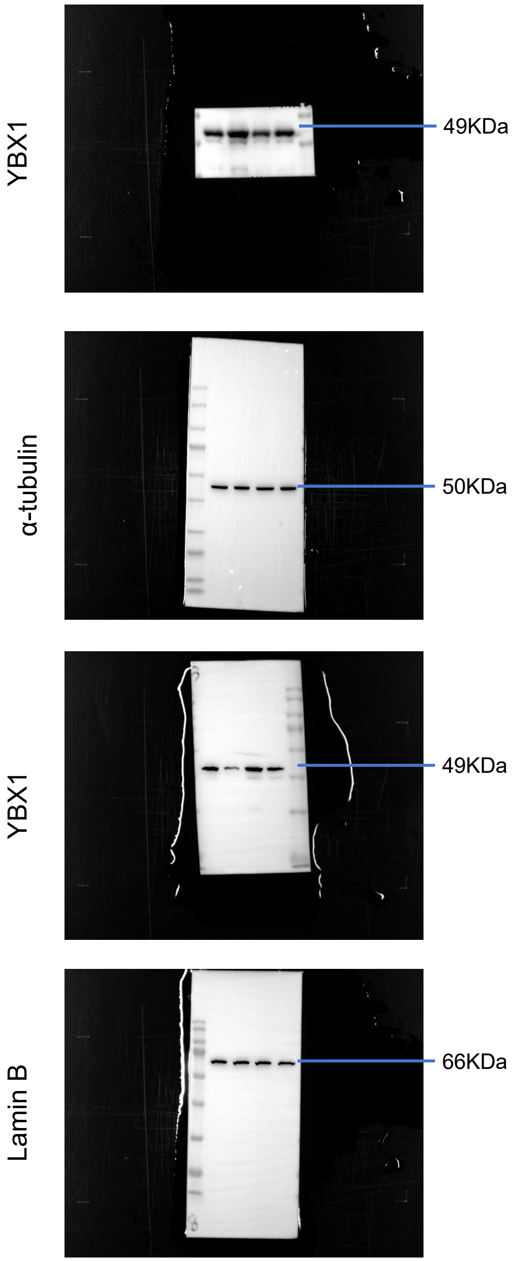

Figure 7B

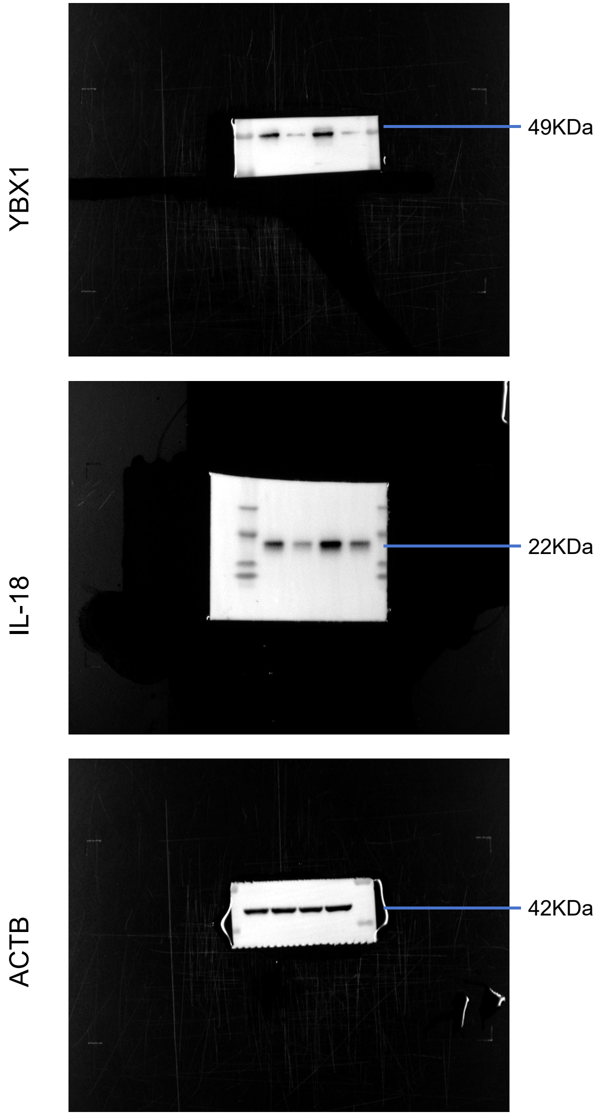

Supplementary Figure 2B

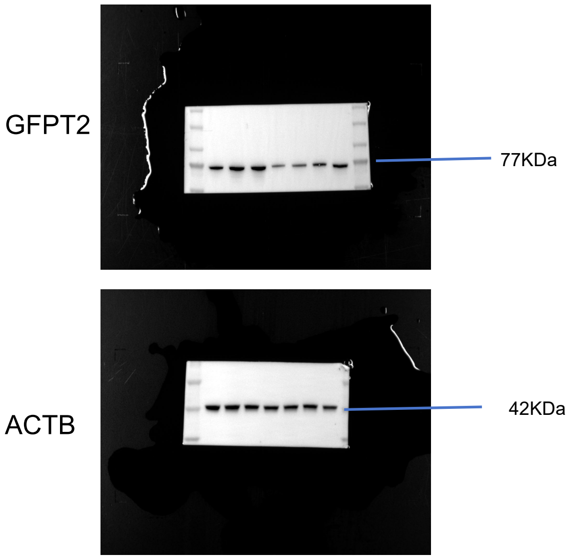

Supplementary Figure 2E

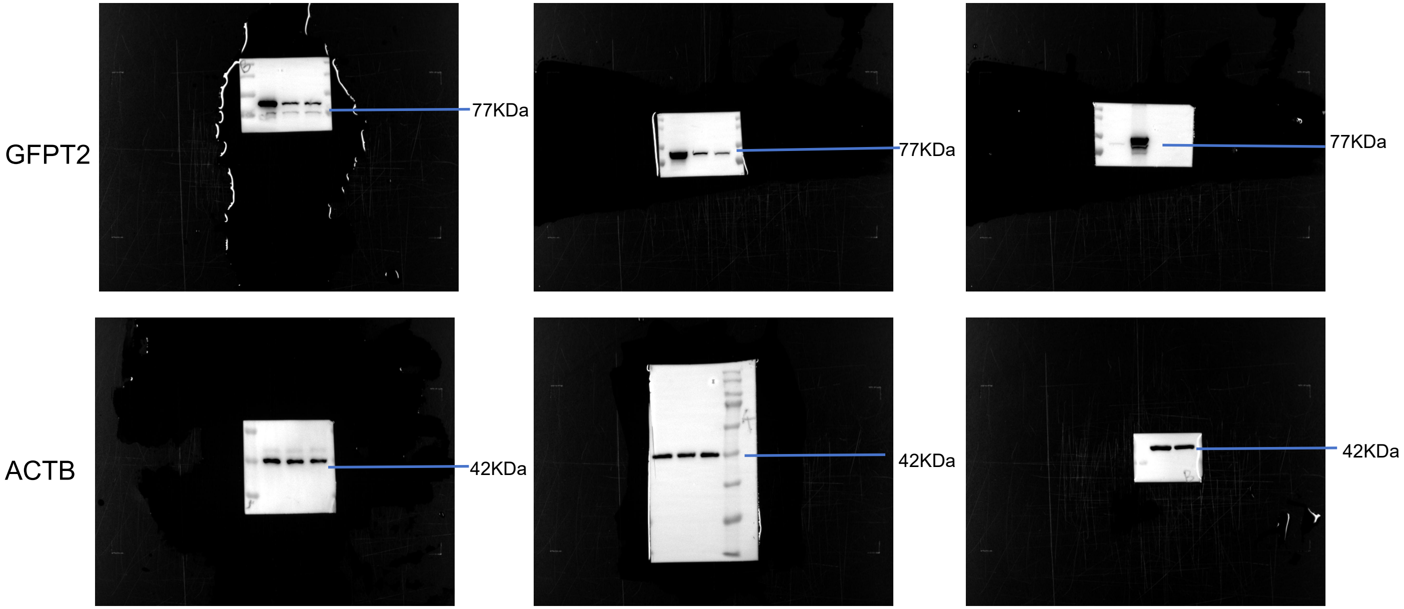

Supplementary Figure 3F

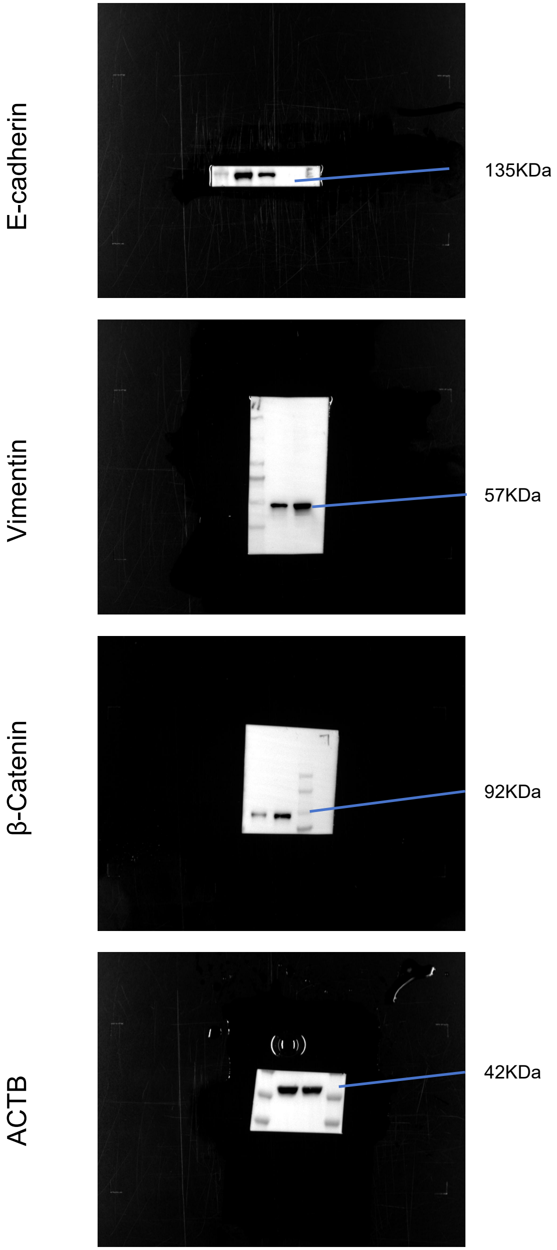

Supplement: Supplementary file 4 — Original Data File [file 41419_2024_6589_MOESM4_ESM.pdf]
